# A Comprehensive Toolbox for Genome Editing in Cultured *Drosophila melanogaster* Cells

**DOI:** 10.1534/g3.116.028241

**Published:** 2016-04-13

**Authors:** Stefan Kunzelmann, Romy Böttcher, Ines Schmidts, Klaus Förstemann

**Affiliations:** Department of Biochemistry, Gene Center, Ludwig-Maximilians-Universität München, D-81377, Germany

**Keywords:** cas9-CRISPR, *Drosophila melanogaster*, homology-directed editing, epitope tag, genome functionalization

## Abstract

Custom genome editing has become an essential element of molecular biology. In particular, the generation of fusion constructs with epitope tags or fluorescent proteins at the genomic locus facilitates the analysis of protein expression, localization, and interaction partners at physiologic levels. Following up on our initial publication, we now describe a considerably simplified, more efficient, and readily scalable experimental workflow for PCR-based genome editing in cultured *Drosophila melanogaster* cells. Our analysis at the *act5C* locus suggests that PCR-based homology arms of 60 bp are sufficient to reach targeting efficiencies of up to 80% after selection; extension to 80 bp (PCR) or 500 bp (targeting vector) did not further improve the yield. We have expanded our targeting system to N-terminal epitope tags; this also allows the generation of cell populations with heterologous expression control of the tagged locus via the copper-inducible *mtnDE* promoter. We present detailed, quantitative data on editing efficiencies for several genomic loci that may serve as positive controls or benchmarks in other laboratories. While our first PCR-based editing approach offered only blasticidin-resistance for selection, we now introduce puromycin-resistance as a second, independent selection marker; it is thus possible to edit two loci (*e.g.*, for coimmunoprecipitation) without marker removal. Finally, we describe a modified FLP recombinase expression plasmid that improves the efficiency of marker cassette FLP-out. In summary, our technique and reagents enable a flexible, robust, and cloning-free genome editing approach that can be parallelized for scale-up.

Genome editing with the help of the CRISPR/*cas* system has become an indispensable part of our toolbox for molecular biology. The CRISPR “revolution” has shifted our attention from cloning work in the context of plasmids or bacterial artificial chromosomes to the modification of genes in their chromosomal context. The creation of a defined DNA double-strand break can either be followed by erroneous repair (end-joining activities), leading to targeted mutagenesis, or by homology-directed repair. The latter offers the possibility to introduce an experimentally provided, custom-modified homologous recombination (HR) donor into a desired locus (Doudna and Charpentier 2014). Various methods that combine programmable cleavage of DNA by CRISPR/*cas9* with introduced HR donors in cell culture have been described (*e.g.*, Bassett *et al.* 2014; Bottcher *et al.* 2014; Byrne *et al.* 2014; Fetter *et al.* 2015; Fu *et al.* 2014a; Li *et al.* 2014; Wyvekens *et al.* 2015). These strategies can be broadly grouped according to the particular type of HR donor material employed: Cloned homology arms, single-stranded synthetic oligonucleotides, or PCR-products with flanking homology regions. We have previously presented a protocol for cultured *Drosophila* *melanogaster* cells that employs PCR to generate both an expression cassette for the *cas9*-programming sgRNA and HR donors for selectable genome modification. In this publication, we describe several technical improvements to our original protocol, two experimental extensions (N-terminal epitope tags and an independent selection marker), and an improved FLP recombinase expression vector. In addition, we provided detailed protocols for use in the laboratory as Supplemental Material, File S1 and File S2.

We present the *act5C*-gene as an easily detectable and quantifiable positive control (via a C-terminal GFP-tag, see Figure 1) that may help other researchers set up the technology in their own labs. With an inverted microscope equipped with fluorescence illumination and a GFP filter set, the first Act5C-GFP positive cells can already be seen about 3 d after the initial transfection. Their enrichment during the selection procedure can also be observed. This model gene has allowed us to optimize a series of parameters (see Figure 2) in our approach to make the method highly efficient yet cost-effective.

**Figure 1:**
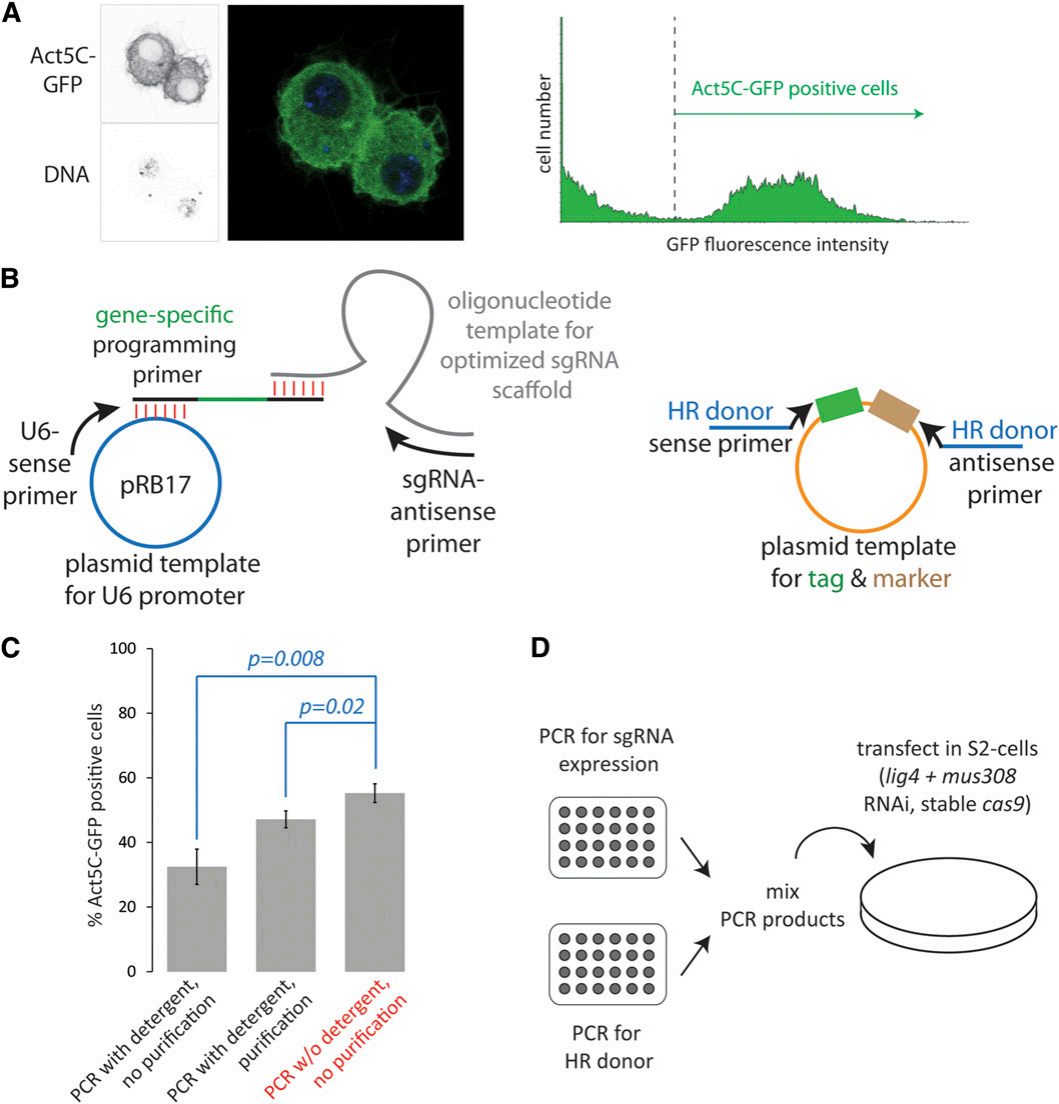
Optimization of technical aspects for the genomic tagging approach. (A) The model locus *act5C* can be efficiently tagged with *e.g.*, GFP. This allows for easy quantification by fluorescence microscopy or flow cytometry. (B) PCR is used to now directly fuse the optimized sgRNA sequence with the U6 promoter; this has allowed a simpler PCR protocol and eliminates T7 *in vitro* transcription. For completeness, the HR donor PCR is also depicted schematically on the right. (C) Commercial PCR buffers contain detergents that interfere with transfection (average ± SD, *n* = 3 biological replicates). If PCR is performed in detergent-free buffer, no purification step is necessary. Both the U6-sgRNA expression fragment and the HR donor were amplified in the respective buffer system. The experiment was performed with knockdown of *lig4* only. (D) The experimental workflow could be simplified with direct U6 fusion and detergent-free buffer. Essentially, it suffices to perform two PCR amplifications, then mix and transfect the products. If the recipient cells do not express *cas9*, a *cas9*-encoding plasmid can be transfected along with the PCR products. GFP, green fluorescent protein; HR, homologous recombination; PCR, polymerase chain reaction; SD, standard deviation; sgRNA, single guide RNA.

**Figure 2:**
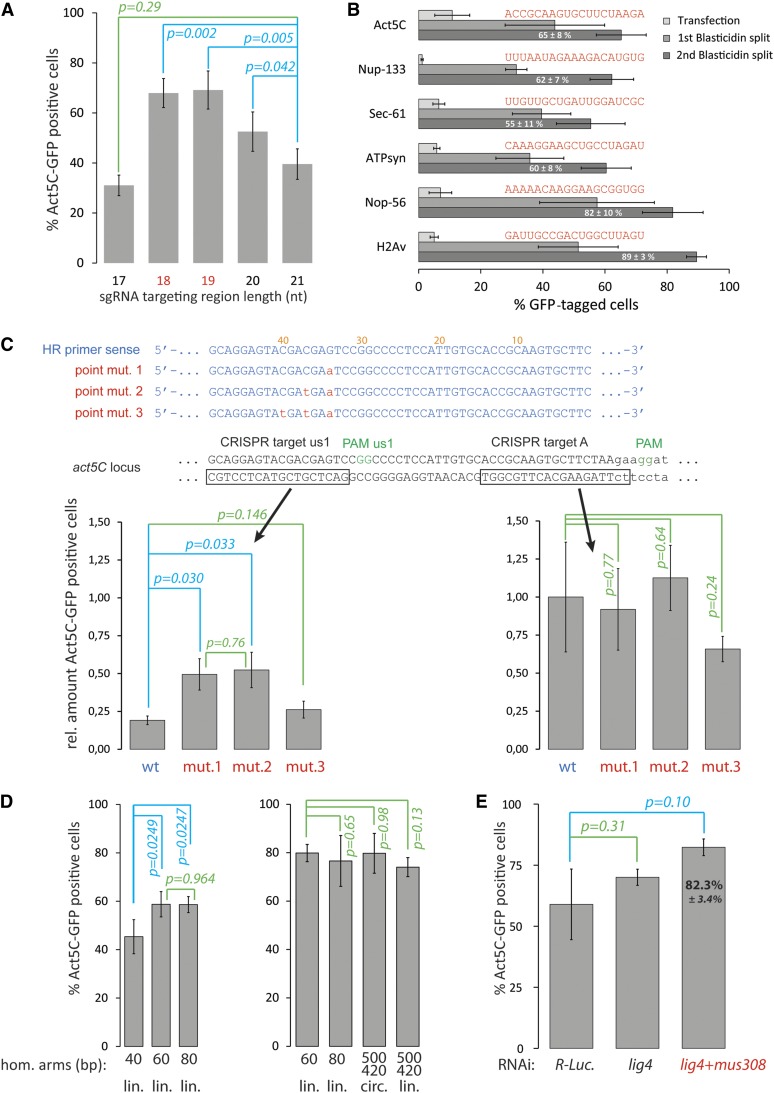
*In vivo* optimization of the tagging approach using the Act5C model locus and flow cytometry-based quantification of the Act5C-GFP tagging success. (A) The optimal length of the target site complementary region of the sgRNA appears to be 18–19 nt (not counting the first G provided by the U6-promoter, see detailed protocol); values depicted are the average ± SD, *n* = 4 biological replicates, *p*-values: *t*-test. This is consistent with the observations made by others (see text for references). (B) We extended our efforts to provide quantitative information about the tagging success rates to five other loci. Shown are the GFP-positive cells detected one week after transfection, upon one round of Blasticidin-selection and after the second round of Blasticidin-selection; depicted are the average values ± SD, *n* = 4 biological replicates. The expression levels of the tagged target loci can be estimated according to the mean intensity of the GFP-signal among the positive cell population (arb. units, see also Figure S1): Act5C-GFP = 293 ± 42, H2Av-GFP = 47 ± 2, Nop56-GFP =19 ± 2, Sec61-GFP = 18 ± 2, ATPsyn-GFP = 16 ± 1, Nup133-GFP = 5 ± 0.3; the corresponding sgRNA sequences are indicated above the respective bars. (C) If an optimally placed GG dinucleotide PAM (*i.e.*, a CRISPR target site that will be destroyed upon integration of the HR donor) cannot be found, the *cas9* cleavage would also occur within the affected homology arm. A single point mutation is sufficient to recover much of the tagging efficiency (left panel, average ± SD, *n* = 3 biological replicates, *p*-values: *t*-test). If cleavage occurs at the optimal position, one or two point mutations do not alter tagging efficiency (right panel, average ± SD, *n* = 3 biological replicates, *p*-values: *t*-test). Note that the values shown represent the initial targeting efficiency. Efficiencies were normalized to the average number of GFP-positive cells obtained when cleavage occurred at the standard site and the homology region did not contain any point mutations (right panel, “wt” bar). The error bars thus reflect both the biological variation for Act5C-GFP tagging and the technical variation of transfection efficiency between replicates. This experiment was performed without depletion of *lig4* and *mus308* because repair via end-joining activities may be required for mutagenesis after cleavage by *cas9*. (D) Left panel: To determine the optimal length of the homology region contained in the PCR primers, we performed the Act5C-GFP tagging experiment with 40, 60, and 80 bp homology regions on either side. While 60 bp of homology length did enhance the efficiency relative to 40 bp, further extension to 80 bp did not increase the efficiency (average ± SD, *n* = 4 biological replicates, *p*-values: *t*-test). We thus recommend using 60 bp of target homology on either side of the HR donor PCR product. Right panel: We compared PCR-based HR donors with longer homology regions contained in a targeting vector (obtained by modification of the PCR template vector). Note that for this panel, a concomitant *lig4/mus308* knockdown was performed (average ± SD, *n* = 3 biological replicates, *p*-values: *t*-test; see also Figure S2). (E) Concomitant knockdown of *lig4* and *mus308* improves the recovery of correctly genome-modified cells. The control knockdown was dsRNA directed against *Renilla* *reniformis* luciferase. The efficiency in this experiment was 82 ± 3% of Act5C-GFP-positive cells for the *lig4/mus308* knockdown (average ± SD, *n* = 3 biological replicates, *p*-values: *t*-test). See also the left and right panels of Figure 2D. CRISPR, Clustered regularly-interspaced short palindromic repeat; GFP, green fluorescent protein; HR, homologous recombination; PAM, protospacer adjacent motif; PCR, polymerase chain reaction; RNAi, RNA interference; SD, standard deviation; sgRNA, single guide RNA; wt, wild-type.

Although cell culture is clearly a reduced system compared to *in vivo* work, it can complement *Drosophila* experimental strategies with respect to cell-autonomous phenomena. In fact, *Drosophila* S2-cells can be cultured essentially without dedicated equipment; while sterile working techniques are of course aided significantly by laminar flow hoods, the cells can also be handled with proper care on a clean laboratory bench. Since the popular Schneider’s medium contains pH-buffering substances, cell culture incubators with CO_2_-control are not required. If necessary, the cells can even be grown in a 25° incubator together with fly stocks (*e.g.*, inside a plastic box). The technique described in this manuscript is thus inexpensive to set up (only requiring standard molecular biology equipment) and can be employed in essentially any fly lab. Our stable *cas9*-expressing cell line is a convenient simplification, but cotransfection of a *cas9* expression plasmid is also possible (Bottcher *et al.* 2014). All plasmids described in this manuscript have been deposited at Addgene (see Table 1 for a full list of plasmid reagents).

**Table 1:** Currently available plasmids for the genomic tagging procedure

		Blasticidin Resistance		Puromycin Resistance		Inducible Blasticidin Resistance	
Type	Tag	Name	Addgene Number	Name	Addgene Number	Name	Addgene Number
C-terminal tag							
	eGFP	pMH3	52528	pSK23	72851	—	—
	twin-Strep	pIW1	52530	pSK24	72852	—	—
	2 × Flag	pMH4	52529	pSK25	72853	—	—
	TEV-V5(long)	—	—	pSK32	72854	—	—
	TEV-V5(short)	pKF296	74773	pKF297	74774	—	—
	PhiC31 attP site	pSK15	72885	pSK41	74886	—	—
Targeting vector	Act5C-eGFP	pIS1	74887	—	—	—	—
N-terminal tag							
	Base vector	pRB36	72861	pRB35	72866	pKF292	72856
	3 × Flag	pRB34	72862	pRB33	72867	pKF293	72857
	1 × Strep	pRB32	72863	pRB31	72868	pKF294	72858
	Strep-His_8_-Strep-TEV	pRB38	72864	pRB37	72869	pKF290	72859
	eGFP	pRB40	72865	pRB39	72870	pRB30	72860
sgRNA expression	snRNA:U6:96Ac prom.	pRB17	52527	—	—	—	—
FLP-recombination	NLS-FLP (P2->S)	pKF295	72871	—	—	—	—

sgRNA, single guide RNA.

## Materials and Methods

### Molecular biology methods

All PCR primer sequences are listed in Table S1. The Act5C-GFP targeting vector was constructed by inserting PCR-amplified homology regions of 500 bp (upstream) and 420 bp (downstream) into the vector pMH3 using the *Not*I/*Xho*I (upstream) and *Cla*I/*Hin*dIII (downstream) restriction sites. Note that, for the downstream homology region, an internal *Hin*dIII site in the homologous sequence served as a cloning site. The improved FLP-recombinase expression plasmid was constructed by amplifying the FLP sequence using primers Bam_NLS_FLPe_sense and Not_FLP_as, then digesting the PCR product with *Bam*HI/*Not*I followed by ligation into correspondingly cleaved pMH5. Candidate clones were sequenced and pKF295 was retained as it carries the desired P(2)->S mutation and the NLS. In addition, the corresponding FLP protein carries a I(377)->L substitution likely due to PCR mutagenesis, which we estimate to be without major consequences. Plasmids for N-terminal HR donor templates were generated through a combination of oligonucleotide synthesis (details available on request), PCR, and restriction enzyme cloning. All oligonucleotides for this manuscript were ordered from Eurofins Genomics (Ebersberg, Germany). The *copia*-puromycin resistance cassette was ordered as a synthetic gBlock fragment (IDT, Coralville).

### Cell culture

Culture of *Drosophila* cells in Schneider’s medium (Bio & Sell, Nürnberg, Germany) supplemented with 10% FBS and penicillin/streptomycin (Life Technologies) and transfection conditions were as previously described (Bottcher *et al.* 2014; Shah and Forstemann 2008). Details on the transfection of PCR products are given in the detailed protocol provided in File S1 and File S2. GFP expression was quantified using a Becton-Dickinson FACScalibur flow cytometer; data analysis was performed with Flowing Software 2.0 (http://www.flowingsoftware.com) using a two-dimensional plot (SSC and GFP fluorescence) to separate nonfluorescent from GFP-positive cells. Fluorescence microscopy images were acquired on a Leica DM IRE2 confocal microscope. Induction of the *mtnDE* promoter was achieved by adding CuSO_4_ from a sterile 100 mM stock solution directly into the culture medium (final concentrations between 30 µM and 1 mM). The effective concentrations are likely somewhat lower since amino group-containing components from the medium form complexes with copper ions (visible as a change to a slight blue color with increasing concentrations). The conditions for induction should therefore be verified if serum-free culture medium is employed.

### Data availability

This article is accompanied by detailed experimental protocols provided in File S1 and File S2. We maintain these protocols and the potentially updated versions can be downloaded from our web-page: http://www.foerstemann.genzentrum.lmu.de/protocols.

The described plasmids are available at Addgene (see Table 1 for accession numbers) and we will provide the S2 cell line with stable *cas9* expression upon request. This cell line is based on our laboratory stock of S2 cells. The entire procedure described here can easily be transferred to other cell lines or engineered *cas9* protein variants by cotransfection of a *cas9* expression plasmid (*e.g.*, pRB14). This strategy is slightly less convenient but, with PCR product-based sgRNA delivery, reaches nearly identical efficiencies (see Figure 5B in Bottcher *et al.* 2014).

The authors state that all data necessary for confirming the conclusions presented in the article are represented fully within the article.

## Results and Discussion

### Act5C-GFP as a positive control locus and model gene for optimization

Since our original publication, we have strived for an improved and simplified genome editing workflow. An important tool to assess beneficial changes in the protocol is a model locus that allows for single cell-based detection of the desired homology-directed modification even before antibiotic selection. We have made extensive use of the C-terminal addition of GFP to the *act5C* gene for this purpose. The GFP modification appears to be well tolerated in cells and can be scored by fluorescence microscopy or flow cytometry as early as 3 d after the transfection (Figure 1A). We also found this to be a very helpful positive control to establish the workflow and to routinely confirm that cell growth and transfection conditions were appropriate.

### Improved sgRNA primer design and direct U6 promoter-sgRNA fusion

The use of DNA-based sgRNA expression constructs, rather than *in vitro* transcribed RNA, has greatly simplified the experiment and work in our laboratory now relies exclusively on this approach. In our first publication (Bottcher *et al.* 2014), we had used the T7-promoter present on the *in vitro* transcription template to generate a U6-promoter fusion product by overlap extension PCR. Since we essentially abandoned *in vitro* transcription of the sgRNA, we designed a new primer template that results in a direct fusion of the sgRNA sequence to the U6:96Ac promoter contained on plasmid pRB17 (Bottcher *et al.* 2014). Furthermore, we exchanged the sgRNA scaffold oligonucleotide for an optimized version developed by the Weissman lab (Chen *et al.* 2013). Generation of an sgRNA template by PCR is now a highly reliable, single-step PCR (Figure 1B). Since the gene-specific sequence in the oligonucleotide that bridges the U6-promoter with the sgRNA scaffold does not participate in any annealing step during PCR, multiple different sgRNA amplifications can be performed in parallel under identical conditions. We recommend including a control amplification without addition of the “programming” gene-specific oligonucleotide; this will reveal if cross-contaminations have occurred.

### Use of detergent-free PCR buffer eliminates the need for purification

PCR products generated in commercial reaction buffers need to be purified in order to reach acceptable transfection efficiencies. We hypothesized that the detergent contained in the commercial buffers might be the reason. Thus, we performed our PCR in self-made reaction buffer that lacks detergent and transfected the products directly, *i.e.*, without any purification, enrichment, or precipitation step. As shown in Figure 1C, the success rate was slightly higher than the one obtained with column-purified material. Perhaps more importantly, this improvement was reached with a greatly simplified experimental workflow (Figure 1D).

### An optimal sgRNA length of 18–19 nucleotides

We previously demonstrated that sgRNA length can be varied (Bottcher *et al.* 2014), and others (*e.g.*, Fu *et al.* 2014b) have demonstrated that truncated sgRNAs provide improved efficacy and specificity for genome editing. Because there is only a narrow window for optimal DNA cleavage sites in our genomic tagging strategy, few (if any) sgRNA alternatives exist and strategies that improve *cas9* cleavage specificity and/or efficiency are thus beneficial. We optimized the length of the target-matching region in the sgRNA and tested this using our Act5C-GFP system. Consistent with previous observations (Fu *et al.* 2014b), we found that a length of 18–19 nucleotides (nt) appears to give optimal efficiency. We did not test whether cleavage specificity was concomitantly improved (as has been reported by others).

It is conceivable, if not obvious, that genome editing efficiencies may vary among targeted sites. We extended our flow cytometry-based, quantitative analysis of C-terminal GFP-tagging efficiency from Act5C (CG4027, X Chr.) to five additional loci: Nup-133 (CG6958, third Chr.), Sec-61 α (CG9539, second Chr.), ATP synthase β (CG11154, fourth Chr.), Nop56 (CG13849, third Chr.), and H2Av (CG5499, third Chr.). These loci were chosen because of their specific localization pattern, allowing for microscopic verification (see Figure S1), and because their expression levels allowed us to quantify the tagging success via flow cytometry. We did not observe any anomalies in cell morphology or grossly altered proliferation rates caused by the fusion proteins. We could obtain efficiencies between 55–89% after two rounds of Blasticidin selection (Figure 2B). This demonstrates that high targeting success rates are not limited to the Act5C locus. However, the number of loci we now present is still limited and their selection was not random. We suggest considering the observed success rates of 55–89% as the range that can be expected for modification of “permissive” loci. In addition to potential chromatin structure-dependent hindrances for DNA cleavage and repair, appending an epitope tag to cellular proteins may interfere with their function. Our approach modifies the genomic allele(s); dominant-negative or haploinsufficiency effects may thus also reduce the yield of cells if the desired modification leads to protein malfunction.

Are there any particular sequence features that determine the efficiency of sgRNA cleavage? This is currently a matter of intense debate and several publications have addressed this issue with diverging conclusions (*e.g.*, Housden *et al.* 2015; Moreno-Mateos *et al.* 2015; Ren *et al.* 2014). We did not find any particular sequence feature that most or all of the six sgRNAs we tested in Figure 2B have in common. This is not too surprising, given the small number of loci, but it suggests that sequence optimization of the sgRNA may only have limited benefit. For the purpose of introducing an epitope-tag at the N- or C-terminus of a protein, the position where *cas9*-dependent cleavage must occur is rather narrowly defined; cleavage further away from the start- or stop-codon rapidly reduces overall efficiency (see Figure 2C). Therefore, if sequence-based sgRNA selection rules are in clear conflict with the need to cleave close to the desired integration site, we suggest choosing the sgRNA sequence in favor of the best cleavage position. Obviously, repetitive sequences that are present in several instances throughout the genome should nonetheless be avoided.

### Single point mutations suffice to prevent cleavage at target sites within homology regions

Sometimes the ideal situation, where the *cas9* target site is disrupted upon insertion of the HR donor, cannot be reached because an appropriately placed GG PAM is absent. In this case, the *cas*9 cleavage site will be present, both in the genomic sequence close to the desired integration site and also in one of the flanking homology regions in the HR donor. To prevent cleavage of the latter prior to integration, or subsequent mutagenesis of the edited locus, a silent point mutation should be introduced in the HR donor. We simulated this situation by deliberately cleaving upstream of the *act5C* stop codon. Perhaps not surprisingly, moving the cleavage site 20 bp away from the desired integration site reduced the efficiency of our genome editing procedure; most likely, this is because the effective length of one homology arm is correspondingly shortened. This efficiency reduction was particularly obvious if HR donors with wild-type sequence were used, presumably because the homology arm was cleaved off prior to integration or because of secondary mutagenesis after integration. A single point mutation within the corresponding HR donor primer was sufficient to restore efficiency; increasing the number of mismatches to the sgRNA sequence did not lead to further improvements (Figure 2C).

### Homology arm lengths of 60 nt suffice for optimal efficiency

Our approach relies on the incorporation of homology arms into PCR primers during oligonucleotide synthesis. This is very convenient but also represents the most expensive reagent in our protocol. Therefore, it is imperative to minimize the length of these homology arms. We had previously demonstrated that 26 bp can suffice for sequence-specific integration, but the efficiency was clearly lower than with 80 bp. We have now compared homology arm lengths of 40, 60, and 80 bp (Figure 2D, left panel). According to our results, homology arm lengths of 60 bp are sufficient for optimal efficiency. While a shortening of the homology to 40 bp clearly reduced the efficiency, an extension to 80 bp did not result in further improvement. To apply this analysis to even longer homology arms, we generated an Act5C-GFP targeting vector (pIS1) by sequentially cloning homology arms of 500 bp upstream and 420 bp downstream homology into our pMH3 template vector. We compared this targeting vector in both circular and linearized form to PCR products with 60 and 80 bp of flanking homology (Figure 2D, right panel). Increasing the homology arm length from 60/60 to 500/420 did not result in an increased overall efficiency (Figure 2D, right panel) or initial recombination rate (Figure S2). Note that in Figure 2D only the right panel was generated with a concomitant *lig4/mus308* knockdown (see below), whereas the left panel was generated with only a *lig4* knockdown.

Taken together, we recommend a homology arm length of 60 bp on each side for maximal efficiency; nonetheless, shorter homology arms (*e.g.*, 40 bp each) may be an acceptable compromise to reduce the costs in large-scale efforts if a somewhat lower efficiency can be tolerated. It is possible that extending the homology regions beyond 500 bp will result in even higher targeting rates. Our interpretation is, however, that beyond a size of 60 bp, the homology arm length is not the limiting factor in this genome editing protocol.

### Improved efficiency due to combined knockdown of lig4 and mus308

The selection of genome-modified cells by resistance to the antibiotic blasticidin-S has allowed us to significantly enrich the desired outcome in the final cell population. However, cells may also become resistant if the HR donor construct integrates spontaneously at a random position within the genome. We refer to these events as “off-site” integrations, to distinguish them from the “off-target” cleavage that may occur with the *cas9* nuclease. As originally demonstrated with mutant flies (Carroll *et al.* 2008), transient depletion of DNA ligase 4 by RNAi reduces these off-site integrations and increases the yield of desired cells (Bottcher *et al.* 2014). This beneficial effect can be further enhanced by a concomitant depletion of the DNA polymerase θ ortholog *mus308*, an enzyme that is important during microhomology-mediated end joining (Figure 2E). In experiments where a concomitant knockdown of *lig4* and *mus308* was combined with a 19 nt optimal-length sgRNA and 60 nt long homology arms, we could reproducibly achieve an Act5C-GFP targeting rate of > 65% among the selected population (Figure 2, B and E and unpublished results). This is likely sufficient for many applications that rely on a gain-of-function approach, such as the introduction of epitope tags or fluorescent markers. Note that some of the experiments in this manuscript (Figure 1C and Figure 2, A and D, left pane) were performed using only a *lig4* knockdown. The parameters that we optimized in these tests are unlikely to be affected by the additional *mus308* knockdown. In principle, a combination of *lig4* and *mus308* alleles should be beneficial for homology-directed genome editing in injected fly embryos or other organisms as well; however, we observed that the two genes display synthetic lethality at higher dsRNA doses.

### Extension of the approach to N-terminal tags

**Figure 3:**
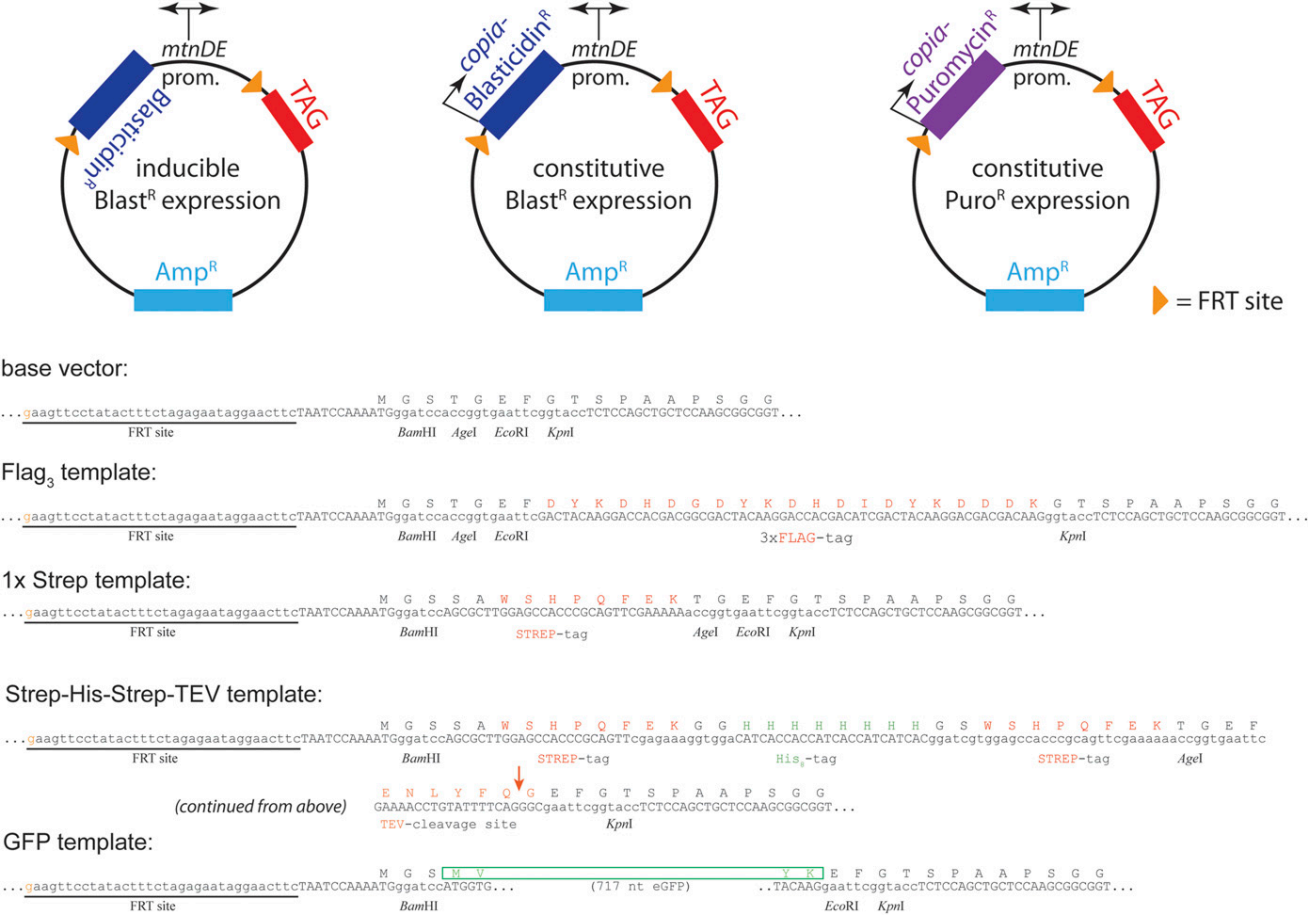
Template vectors for N-terminal tagging. Top row: schematic representation of the three different vector template series used for N-terminal tagging. In the first series (left), the inducible and bidirectional mtnDE promoter drives the expression of the Blasticidin-resistance gene. We recommend adding CuSO_4_ to a concentration of 100 µM or higher 2 d prior to transfer into selective medium. Constitutive expression (driven by the *copia* promoter) of the Blasticidin or Puromycin resistance genes occurs, respectively, when the second (center) or third (right) series of templates is employed. Note that a single HR-containing primer set can be used with any of these templates. Bottom: Sequence details of the different N-terminal tags for which we developed template vectors. Amp^R^, ampicillin resistance; Blast^R^, blasticidin resistance; FRT, Flp recombinase target; GFP, green fluorescent protein; His, histidine; HR, homologous recombination; TEV, tobacco etch virus protease cleavage site.

We extended our approach to the introduction of N-terminal protein tags. To this end, a new template vector design had to be developed that also comprises a promoter for heterologous expression of the tagged gene. This is necessary because, in the case of an N-terminal tag, the selection cassette separates the endogenous promoter from the gene body. We chose the inducible, bidirectional *mtnDE* promoter for this purpose. Induction is possible by adding *e.g.*, CuSO_4_ to the growth medium and the promoter can drive expression of the selection marker and the tagged protein concomitantly. In addition, we combined the *mtnDE*-driven expression of the tagged gene with constitutively active marker cassettes, to select modified cells without the need for induction of the tagged gene. This may be helpful if expression of the resulting fusion gene is toxic (*e.g.*, due to overexpression, generation of N-terminal truncations, misexpression etc.). As for the C-terminal approach, our vector templates bear constant regions for annealing of the homology-containing targeting primers during PCR. All N-terminal tags and selection constructs can thus be amplified with a single set of HR-containing primers (summarized in Figure 3). We have developed N-terminal vector templates for GFP, the 3 × Flag epitope, a single Strep-tag, and a combined Strep-His_8_-Strep-TEV tag; the latter one can be removed from the purified protein by TEV-cleavage (see Table 1 for an overview of all template vectors currently available at Addgene). The His_8_-sequence allows removal of the cleaved tag from the reaction along with the usually His-tagged TEV protease protein. The tag may also be used for tandem affinity purification (we recommend to start with Ni-NTA beads and use Streptactin beads as the second step). Since the selection cassette is flanked by FRT-sites in all vectors, it can be removed with FLP recombinase to restore expression control by the endogenous promoter. Note that both the N- and C-terminal template vectors can easily be modified, *e.g.*, using restriction enzyme-based cloning to harbor other tags, fluorescent proteins, or elements for genome functionalization. For example, we generated template vectors that allow the introduction of an *attP* target site based on the C-terminal epitope tag template series (see Table 1).

### Heterologous control of gene expression by the mtnDE promoter

**Figure 4:**
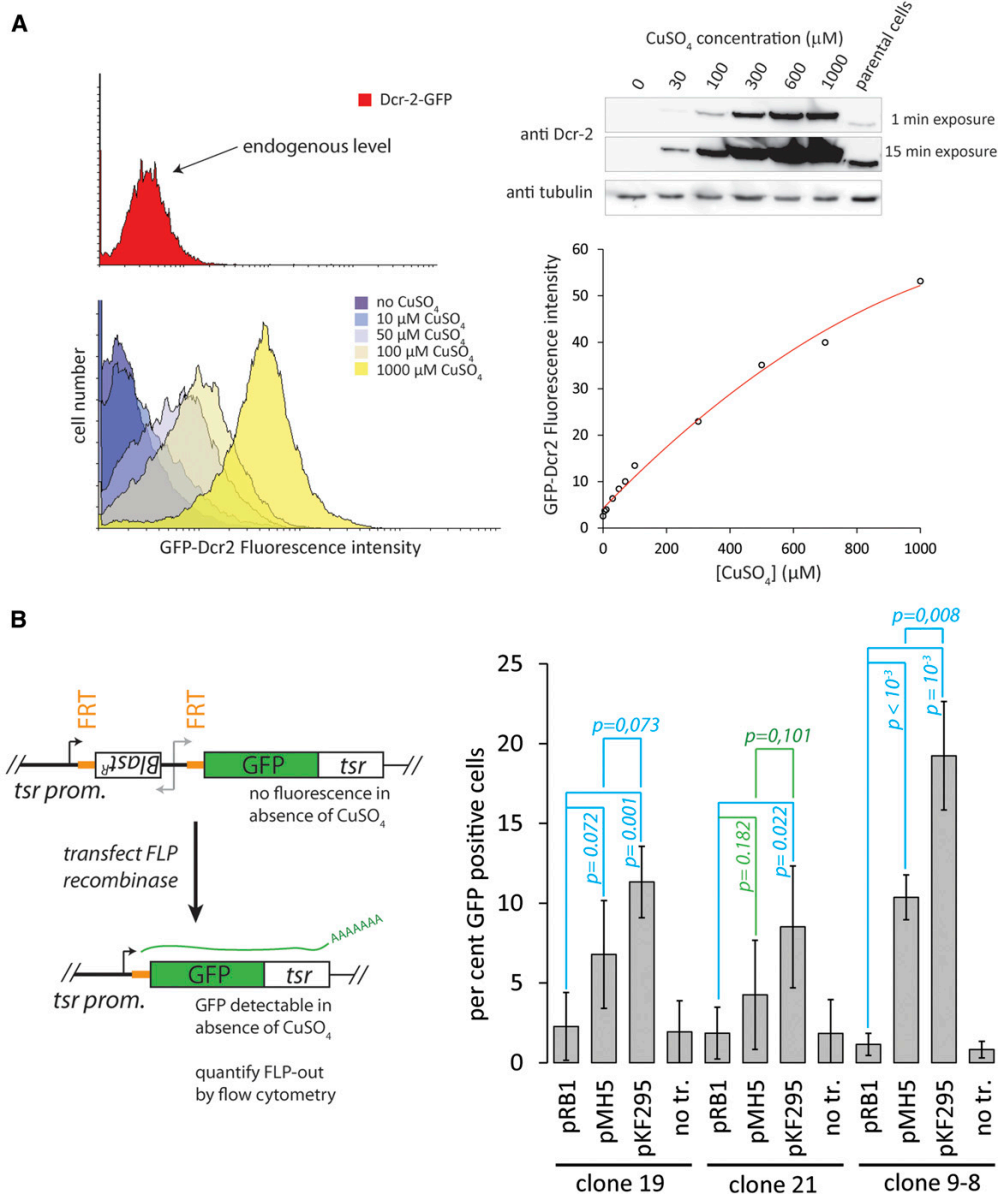
Heterologous control of gene expression with the *mtnDE* promoter and quantification of FLP-out efficiency. (A) We could generate clonal cell populations where all *dcr-2* alleles had been modified to include an N-terminal GFP tag. Prior to marker excision, this allows for heterologous and tunable control of GFP-Dcr-2 expression levels. We quantified the amount of modified Dcr-2 protein per cell by comparing the N-terminal fusions (bottom left) with a C-terminal fusion (top left) that presumably reflects the endogenous level of Dcr-2 expression. We estimate that, in this particular case, a CuSO_4_ concentration of 30–50 µM leads to wild-type expression levels. Western blotting experiments with a monoclonal antibody directed against the endogenous protein confirmed the absence of untagged protein and that CuSO_4_ concentrations between 30–50 µM led to wild-type equivalent expression. The *mtnDE* promoter allows the tuning of expression levels within a roughly tenfold range (see western blot on top right and flow cytometry quantification on bottom right), but absolute levels may vary between genes. (B) An N-terminal GFP-tag at the *twinstar (tsr)* locus served as a test-platform to determine the FLP-out efficiency. The left panel depicts the experimental approach: In the absence of CuSO_4_, no GFP-expression is detectable in cells that harbor one or more edited *tsr* alleles. Upon FLP-mediated recombination between the FRT sites flanking the marker gene and *mtnDE*-promoter, transcription of the fusion gene is again controlled by the endogenous *tsr* promoter and GFP-tsr fluorescence can be measured and quantified by flow cytometry. This is depicted in the right panel for three clonal cell populations carrying modified *tsr* alleles. Untransfected cells and cells transfected with a luciferase-expression plasmid served as controls; we compared our previously published FLP expression vector (pMH5) with an improved version (pKF295). The depicted values are the mean ± SD, *n* = 4 biological replicates (clone 21: 5 biological replicates), *p*-values: *t*-test. FRT, Flp recombinase target; GFP, green fluorescent protein; SD, standard deviation.

If all alleles for a given gene in the S2-cell genome have been modified (see File S1 and File S2 for clonal selection of cells and PCR genotyping strategies), the introduced mtnDE-promoter allows heterologous control over the expression of the targeted gene. As an example, we derived cell lines with N-terminally GFP-tagged Dcr-2 protein. After clonal selection, we could readily identify cells that carried only modified *dcr-2* alleles. These lines allowed us to tune the expression level of Dcr-2; analysis by FACS and western blotting demonstrated that both transcriptional shut-off and overexpression situations can be obtained (Figure 4). We have not extensively characterized the leakiness of the *mtnDE* promoter in the noninduced state, but preliminary results indicate that the transcript levels are comparable to those observed after RNAi (data not shown; this likely also varies according to the genomic position). For simple loss-of-function approaches, RNAi is far easier to apply; however, the mtnDE promoter “alleles” may present an interesting tool to study genetic interaction in combination with RNAi of a second factor. In particular, they may be convenient to create hypomorphic expression levels of essential genes in order to make them amenable for synthetic genetic screens.

### Generation of an improved FLP-recombinase expression vector

We reasoned that if a robustly expressed gene is N-terminally GFP-tagged with our system, then the efficiency of marker-cassette FLP-out can be quantified by measuring the proportion of GFP-positive cells in the absence of CuSO_4_, once expression is again driven by the endogenous promoter. We designed oligonucleotides targeting the *twinstar (tsr)* gene, a highly expressed *Drosophila* homolog of cofilin. Stable and clonal cell populations were selected and flow cytometry was used to verify that all cells in the populations became GFP-positive after CuSO_4_ induction (data not shown). Successful cassette FLP-out could then be measured as the amount of GFP-positive cells following transfection of a FLP-recombinase expression plasmid. The measured GFP-fluorescence intensities were lower than what we had expected based on published *tsr* expression levels; nonetheless, the system allows reliable quantification of the successful FLP-out events. We had previously described an expression vector for wild-type *Saccharomyces cerevisiae* FLP recombinase (pMH5) and noticed that the efficiency of FLP-out was somewhat limited. Improved versions of the FLP recombinase have been described (Buchholz *et al.* 1998) and we appended an N-terminal nuclear localization signal in combination with the proline(2) -> serine substitution to generate plasmid pKF295. Using the GFP-*tsr* test system in three separate clonal cell lines, we found that transfection of pKF295 approximately doubled the amount of GFP-positive cells compared to pMH5 (Figure 4B). In absolute terms, a FLP-out efficiency of 5–10% (pMH5) and 10–20% (pKF295) could be reached. Considering the transfection efficiency of ∼60%, the higher values correspond to about one third (pKF295) and one fifth (pMH5) of the transfected cells.

### Introduction of a second selection cassette

To enable straightforward introduction of a second epitope tag (*e.g.*, for coimmunoprecipitation studies), we developed an independent selection cassette based on the puromycin acetyl transferase gene. Since the commonly used coding sequence of this gene proved to be rather refractory to PCR amplification (likely due to a high GC content), we created a *copia*-Puro resistance cassette as a synthetic gene. This element could be readily amplified by PCR and we have generated alternative versions of most template vectors by exchanging the *copia*-Blast cassette with the *copia*-Puro marker (see Figure 3 and Table 1). As a general proof-of-principle, we show the introduction of a C-terminal twin-Strep tag (pSK24-based, see Table 1) at the Dcr-2 locus (Figure S3). We observed that our S2-cell line is quite sensitive to puromycin; selection works well at a concentration of 0.5 µg/ml of puromycin in Schneider’s medium. Integration of a puromycin construct in a blasticidin-resistant cell line (or vice versa) is efficient and does not require any changes to the protocol (data not shown). We have not quantified the tagging efficiencies with puromycin resistance-based constructs in a manner analogous to the experiments shown, *e.g.*, in Figure 2. The tagging success rates clearly depend on optimal sgRNA length and the extent of homology arms in the HR donor PCR product; since these elements are independent of the marker chosen, we do not expect major quantitative differences between puromycin and blasticidin-based selections.

In principle, the two constructs could also be integrated in parallel rather than sequentially. Indeed, we have been able to recover cells that underwent corresponding multiplex genome editing. However, these cells are very rare in the initial population due to the rather low initial targeting frequency (3–10%). As a consequence, the multiplexed editing approach is not very robust and more time is required until sufficient cells have grown for downstream analysis. We therefore recommend sequential introduction if multiple epitope tags need to be combined within the same cell. If one begins with an inducible blasticidin resistance construct for an N-terminal tag (pKF290-294 or pRB30, see Table 1) and then continues with constitutive puromycin and blasticidin resistance cassettes, up to three epitope tags can be combined without the need to FLP out the marker in between.

### Conclusions

In this publication, we describe a simplified genome editing protocol with increased efficiency, an extension to N-terminal epitope tags and the application of a second, independent selection cassette for our PCR-based genome editing of cultured *Drosophila* cells. Table 1 summarizes all of the template vectors that we have developed to date. The genomic tagging system now offers significantly increased functionality, including the possibility to verify protein–protein interactions via coimmunoprecipitation. The improved primer design to generate U6-sgRNA expression cassettes has made the system much more robust and enables the reliable generation of reagents for many genes in parallel. Due to the use of PCR rather than cloning, our experimental approach is flexible, scalable, and can easily accommodate future changes (*e.g.*, different sgRNA designs, novel epitope tags). We estimate that it should be straightforward to extend our strategy to other *Drosophila* cell culture systems, potentially even to cultured cells from other insect species. Related PCR-based approaches have been described for use in cultured vertebrate cells (Li *et al.* 2014; Stewart-Ornstein and Lahav 2016). We expect that our vector templates can be modified for use beyond insect cells by exchanging the *copia*-promoter used for selection and/or the inducible *mtnDE* promoter with sequences of corresponding functionality in, *e.g.*, vertebrate cells. Perhaps more importantly, it may be possible to transfer the conclusions from our optimization efforts to other cell culture systems as well.

## 

## Supplementary Material

Supplemental Material

## References

[bib1] Bassett A. R. Tibbit C. Ponting C. P. Liu J. L., 2014 Mutagenesis and homologous recombination in *Drosophila* cell lines using CRISPR/Cas9. Biol. Open 3: 42–49. 24326186 10.1242/bio.20137120 PMC3892159

[bib2] Bottcher R. Hollmann M. Merk K. Nitschko V. Obermaier C., 2014 Efficient chromosomal gene modification with CRISPR/*cas9* and PCR-based homologous recombination donors in cultured *Drosophila* cells. Nucleic Acids Res. 42: e89. 24748663 10.1093/nar/gku289 PMC4066747

[bib3] Buchholz F. Angrand P. O. Stewart A. F., 1998 Improved properties of FLP recombinase evolved by cycling mutagenesis. Nat. Biotechnol. 16: 657–662. 9661200 10.1038/nbt0798-657

[bib4] Byrne S. M. Mali P. Church G. M., 2014 Genome editing in human stem cells. Methods Enzymol. 546: 119–138. 25398338 10.1016/B978-0-12-801185-0.00006-4 PMC4408990

[bib5] Carroll D. Beumer K. J. Morton J. J. Bozas A. Trautman J. K., 2008 Gene targeting in *Drosophila* and *Caenorhabditis elegans* with zinc-finger nucleases. Methods Mol. Biol. 435: 63–77. 18370068 10.1007/978-1-59745-232-8_5

[bib6] Chen B. Gilbert L. A. Cimini B. A. Schnitzbauer J. Zhang W., 2013 Dynamic imaging of genomic loci in living human cells by an optimized CRISPR/Cas system. Cell 155: 1479–1491. 24360272 10.1016/j.cell.2013.12.001 PMC3918502

[bib7] Doudna J. A. Charpentier E., 2014 Genome editing. The new frontier of genome engineering with CRISPR-Cas9. Science 346: 1258096. 25430774 10.1126/science.1258096

[bib8] Fetter J. Samsonov A. Zenser N. Zhang F. Zhang H., 2015 Endogenous gene tagging with fluorescent proteins. Methods Mol. Biol. 1239: 231–240. 25408409 10.1007/978-1-4939-1862-1_12

[bib9] Fu Y. Reyon D. Joung J. K., 2014 a Targeted genome editing in human cells using CRISPR/Cas nucleases and truncated guide RNAs. Methods Enzymol. 546: 21–45. 25398334 10.1016/B978-0-12-801185-0.00002-7

[bib10] Fu Y. Sander J. D. Reyon D. Cascio V. M. Joung J. K., 2014 b Improving CRISPR-Cas nuclease specificity using truncated guide RNAs. Nat. Biotechnol. 32: 279–284. 24463574 10.1038/nbt.2808 PMC3988262

[bib11] Housden B. E. Valvezan A. J. Kelley C. Sopko R. Hu Y., 2015 Identification of potential drug targets for tuberous sclerosis complex by synthetic screens combining CRISPR-based knockouts with RNAi. Sci. Signal. 8: rs9. 26350902 10.1126/scisignal.aab3729 PMC4642709

[bib12] Li K. Wang G. Andersen T. Zhou P. Pu W. T., 2014 Optimization of genome engineering approaches with the CRISPR/Cas9 system. PLoS One 9: e105779. 25166277 10.1371/journal.pone.0105779 PMC4148324

[bib13] Moreno-Mateos M. A. Vejnar C. E. Beaudoin J. D. Fernandez J. P. Mis E. K., 2015 CRISPRscan: designing highly efficient sgRNAs for CRISPR-Cas9 targeting *in vivo*. Nat. Methods 12: 982–988. 26322839 10.1038/nmeth.3543 PMC4589495

[bib14] Ren X. Yang Z. Xu J. Sun J. Mao D., 2014 Enhanced specificity and efficiency of the CRISPR/Cas9 system with optimized sgRNA parameters in *Drosophila*. Cell Reports 9: 1151–1162. 25437567 10.1016/j.celrep.2014.09.044 PMC4250831

[bib15] Shah C. Forstemann K., 2008 Monitoring miRNA-mediated silencing in *Drosophila melanogaster* S2-cells. Biochim. Biophys. Acta 1779: 766–772. 18634912 10.1016/j.bbagrm.2008.06.008

[bib16] Stewart-Ornstein J. Lahav G., 2016 Dynamics of CDKN1A in single cells defined by an endogenous fluorescent tagging toolkit. Cell Reports 14: 1800–1811. 26876176 10.1016/j.celrep.2016.01.045 PMC5154611

[bib17] Wyvekens N. Tsai S. Q. Joung J. K., 2015 Genome editing in human cells using CRISPR/Cas nucleases. Curr. Protoc. Mol. Biol. 112: 31.3.1–31.3.18. 26423589 10.1002/0471142727.mb3103s112 PMC4852847

