# Supplementary material for: A Comprehensive Toolbox for Genome Editing in Cultured Drosophila melanogaster Cells
Source: G3 (Bethesda). 2016 Apr 13;6(6):1777–85. doi: 10.1534/g3.116.028241 (PMC4889673; doi:10.1534/g3.116.028241)
Supplement: Supplemental Material [file supp_6_6_1777__index.html]

A Comprehensive Toolbox for Genome Editing in Cultured Drosophila melanogaster Cells — Supplemental Material 

# A Comprehensive Toolbox for Genome Editing in Cultured *Drosophila melanogaster* Cells

## Supplemental Material for Kunzelmann *et al.*, 2016

**Files in this Data Supplement:**

- Table S1 - Oligonucleotide sequences used in this study (sequences are 5' to 3). (.pdf, 69 KB)
- Figure S1 - Live cell confocal images of the GFP-tagged factors from Fig. 2 B in the manuscript. (.pdf, 1721 KB)
- Figure S2 - Comparison of long and short homology arms. (.pdf, 687 KB)
- Figure S3 - Genome editing with the puromycin resistance marker. (.pdf, 225 KB)
- File S1 - Genomic Tagging in *Drosophila* cells (C-terminal constructs). (.pdf, 3.3 MB)
- File S2 - Genomic Tagging in *Drosophila* cells (N-terminal constructs) (.pdf, 3.2 MB)
