# Supplementary material for: A Comprehensive Toolbox for Genome Editing in Cultured Drosophila melanogaster Cells
Source: G3 (Bethesda). 2016 Apr 13;6(6):1777–85. doi: 10.1534/g3.116.028241 (PMC4889673; doi:10.1534/g3.116.028241)
Supplement: Supplemental Material [file supp_g3.116.028241_TableS1.pdf]

Table S1: Oligonucleotide sequences used in this study (sequences are 5' to 3')

**Act5C_CRISPR_17nt**	CCTATTTTCAATTTAACGTCGCGCAAGTGCTTCTAAGAGTTTAAGAGCTATGCTG
**Act5C_CRISPR_18nt**	CCTATTTTCAATTTAACGTCGCCGCAAGTGCTTCTAAGAGTTTAAGAGCTATGCTG
**Act5C_CRISPR_19nt**	CCTATTTTCAATTTAACGTCGACCGCAAGTGCTTCTAAGAGTTTAAGAGCTATGCTG
**Act5C_CRISPR_20nt**	CCTATTTTCAATTTAACGTCGCACCGCAAGTGCTTCTAAGAGTTTAAGAGCTATGCTG
**Act5C_CRISPR_21nt**	CCTATTTTCAATTTAACGTCGGCACCGCAAGTGCTTCTAAGAGTTTAAGAGCTATGCTG

**Act5C_CRISPR_us1**	CCTATTTTCAATTTAACGTCGGCAGGAGTACGACGAGTCGTTTAAGAGCTATGCTG

**CRISPR Nup133**	CCTATTTTCAATTTAACGTCGTTTAATAGAAAGACATGTGGTTTAAGAGCTATGCTG
**CRISPR Sec61**	CCTATTTTCAATTTAACGTCGTTGTTGCTGATTGGATCGCGTTTAAGAGCTATGCTG
**CRISPR_ATP_syn**	CCTATTTTCAATTTAACGTCGCAAAGGAAGCTGCCTAGATGTTTAAGAGCTATGCTG
**CRISPR Nop56**	CCTATTTTCAATTTAACGTCGAAAAACAAGGAAGCGGTGGGTTTAAGAGCTATGCTG
**CRISPR H2Av**	CCTATTTTCAATTTAACGTCGGATTGCCGACTGGCTTAGTGTTTAAGAGCTATGCTG

**CRISPR_Nterm_tsr**	CCTATTTTCAATTTAACGTCGCGAAAAACTACAGGAAAAAGTTTAAGAGCTATGCTG
**CRISPR_Nterm_Dcr-2**	CCTATTTTCAATTTAACGTCGGAGCGAAGATATGGAAGATGGTTTAAGAGCTATGCTG
**CRISPR_Cterm_Dcr2**	CCTATTTTCAATTTAACGTCGGGGCTGGAATCATCATATTGTTTAAGAGCTATGCTG

**Act5C_Ctag_40nt_s**	CGACGAGTCCGGCCCCTCCATTGTGCACCGCAAGTGCTTCGGATCTTCCGGATGGCTCGAG
**Act5C_Ctag_40nt_as**	ATCCCGATCCTGATCCTCTTGCCCAGACAAGCGATCCTTCGAAGTTCCTATTCTCTAGAAAGTATAGGAACTTCCATATG
**Act5C_Ctag_60nt_s**	TGGATCTCCAAGCAGGAGTACGACGAGTCCGGCCCCTCCATTGTGCACCGCAAGTGCTTCGGATCTTCCGGATGGCTCGAG
**Act5C_Ctag_60nt_as**	CCTCCAGCAGAATCAAGACCATCCCGATCCTGATCCTCTTGCCCAGACAAGCGATCCTTCGAAGTTCCTATTCTCTAGAAAGTATAGGAACTTCCATATG
**Act5C_Ctag_80nt_s**	TGTCCACCTTCCAGCAGATGTGGATCTCCAAGCAGGAGTACGACGAGTCCGGCCCCTCCATTGTGCACCGCAAGTGCTTCGGATCTTCCGGATGGCTCGAG
**Act5C_Ctag_80nt_as**	CCTCGACTTCTCCTCCTCCTCCTCCAGCAGAATCAAGACCATCCCGATCCTGATCCTCTTGCCCAGACAAGCGATCCTTCGAAGTTCCTATTCTCTAGAAAGTA<br>TAGGAACTTCCATATG

**Act5C Ctag _sense_point_mutation**	TGTCCACCTTCCAGCAAATGTGGATCTCCAAGCAGGAGTACGACGAATCCGGCCCCTCCATTGTGCACCGCAAGTGCTTCGGATCTTCCGGATGGCTCGAG
**Act5C Ctag_sense_point_mutation 2**	TGTCCACCTTCCAACAAATGTGGATCTCCAAGCAGGAGTACGATGAATCCGGCCCCTCCATTGTGCACCGCAAGTGCTTCGGATCTTCCGGATGGCTCGAG
**Act5C Ctag_sense_point_mutation 3**	TGTCCACCTTACAACAAATGTGGATCTCCAAGCAGGAGTATGATGAATCCGGCCCCTCCATTGTGCACCGCAAGTGCTTCGGATCTTCCGGATGGCTCGAG

**tsr Ntag s**	AAATATATACTCGGCGAAGCAGCAATTTGAGAATTGTGAAAGCGAAAAACTACAGGAAAAGAAGTTCCTATACTTTCTAGAGAATAGGAACTTCCATATG
**tsr Ntag as**	GTGCACAATGACCGGCCGCTTTTACCACCGAGTCTGAATGCGAGTCCCAAGTGTACGTACACCGCCGCTTGGAGCAGCTGGAGA
**Dcr2 Ntag s**	ATCGTAAATTATTTGACTTCAAATTGTAATATCAAAATATCCCGAAGACTCTGTAACCAAGAGCGAAGATGAAGTTCCTATACTTTCTAGAGAATAGGAACTTCCATATG
**Dcr2 Ntag as**	GATTTTGTTAATTATTTTGCAATAAGCGAGAGCGCAAGCCAATTCATTTTACCTTGATTTCCACATCTTCACCGCCGCTTGGAGCAGC
**Dcr-2 Ctag s**	GAAAAGACGATCAAGGTGTACGGATTTGGGAGCAATAAGGACCAGGCCAAGCTATCGGCTGCCAAGCACGCCCTTCAGCAGCTAAGCAAATGCGACGCCGGATCTTCCGGATGGCTCGAG
**Dcr-2 Ctag as**	TCTTCCAAAGGATTACGTCTTGGCTAGAAAGCAAATTCAATGCAGTTTAGCAGAAACTAGACTGGGCTGGAATCATCATAGAAGTTCCTATTCTCTAGAAAGTATAGGAACTTCCATATG

**Nup133 Ctag s**	AGAAACCGTTTCAGTATCTGTTGAAACTGACCTATGAGTATGTGGCCGACATGTTCAAACAGCCAGACGACATGGAACTCGGATCTTCCGGATGGCTCGAG
**Nup133 Ctag as**	TCCAAGAACACCAGCCATCATTTTAAATTCTTAACGTAAACTTTAATAGAAAGACATCTGAGGTGCTTGAGAAGTTCCTATTCTCTAGAAAGTATAGGAACTTCCATATG
**Sec61 Ctag s**	CTGTGACCATCATCTACCAATACTTTGAGATTTTCGTTAAGGAGCAGTCCGAAATGGGCGGCATGGGCACGCTGCTGTTCGGATCTTCCGGATGGCTCGAG
**Sec61 Ctag as**	GCGAGCGTATGGGAAGTATGATGAAGTATTAGTTGTTGCTGATTGGAGCGCAGGTGATCTTCTTGACTGCGAAGTTCCTATTCTCTAGAAAGTATAGGAACTTCCATATG
**ATP_syn Ctag s**	TACCTGAAGTTGCTTTCTACATGGTCGGCCCAATCGAAGAAGTTGTAGAAAAGGCTGACCGCCTGGCAAAGGAAGCTGCCGGATCTTCCGGATGGCTCGAG
**ATP_syn Ctag as**	TATTACAAATTCAATGCATAATATTCAATGATAGATTTGCTGACTTTGAAGATTTTTCCAGTACGCCTATGAAGTTCCTATTCTCTAGAAAGTATAGGAACTTCCATATG
**Nop56 Ctag s**	AAAATGGCAATGGCAACGATGATGGAGAGCCAAAGAAGAAGAAGAAAAAGAAGAACAAAAACAAGGAAGCGGTAGAGGCGGGATCTTCCGGATGGCTCGAG
**Nop56 Ctag as**	AATACAAAAGAAGCATGTAAGTAAAAGATAATAGTGTGGACTTAAACTAAGTTGAATGTAACAGAGACATGAAGTTCCTATTCTCTAGAAAGTATAGGAACTTCCATATG
**H2Av Ctag s**	ACAAGTCGCTGATCGGCAAAAAGGAGGAAACGGTGCAGGATCCGCAGCGGAAGGGCAACGTCATTCTGTCGCAGGCCTACGGATCTTCCGGATGGCTCGAG
**H2Av Ctag as**	CTTGTCTCTGCTGAAATCTGAATTAAACATTAGTGTTGCATGTTTCGAAGGCGTCCGATTGCCGACTGGCGAAGTTCCTATTCTCTAGAAAGTATAGGAACTTCCATATG

**Bam_NLS_FLPe_sense**	ACGGATCCTCCAAAATGGTGCCAAAGAAAAAGCGTAAGGTCTCGATGTCACAATTTGGTATATTATGTAAAAC
**Not_FLP_as**	ATGCGGCCGCTTATATGCGTCTATTTATGTAGGATGAAAG

**Act5C HR donor us sense**	AAAgcggccgcTCCGTGACATCAAGGAGAAG
**Act5C HR donor us antisense**	TTTctcgagCCATCCGGAAGATCCGAAGCACTTGCGGTGCACAA
**Act5C HR donor ds sense**	AAAatcgatGAAGGATCGCTTGTCTGGGC
**Act5C HR donor ds antisense**	TTTaagcttCTTTTTGCGCTTTGGGAAAT
