# Supplementary material for: A Comprehensive Toolbox for Genome Editing in Cultured Drosophila melanogaster Cells
Source: G3 (Bethesda). 2016 Apr 13;6(6):1777–85. doi: 10.1534/g3.116.028241 (PMC4889673; doi:10.1534/g3.116.028241)
Supplement: Supplemental Material [file supp_g3.116.028241_FigureS1.pdf]

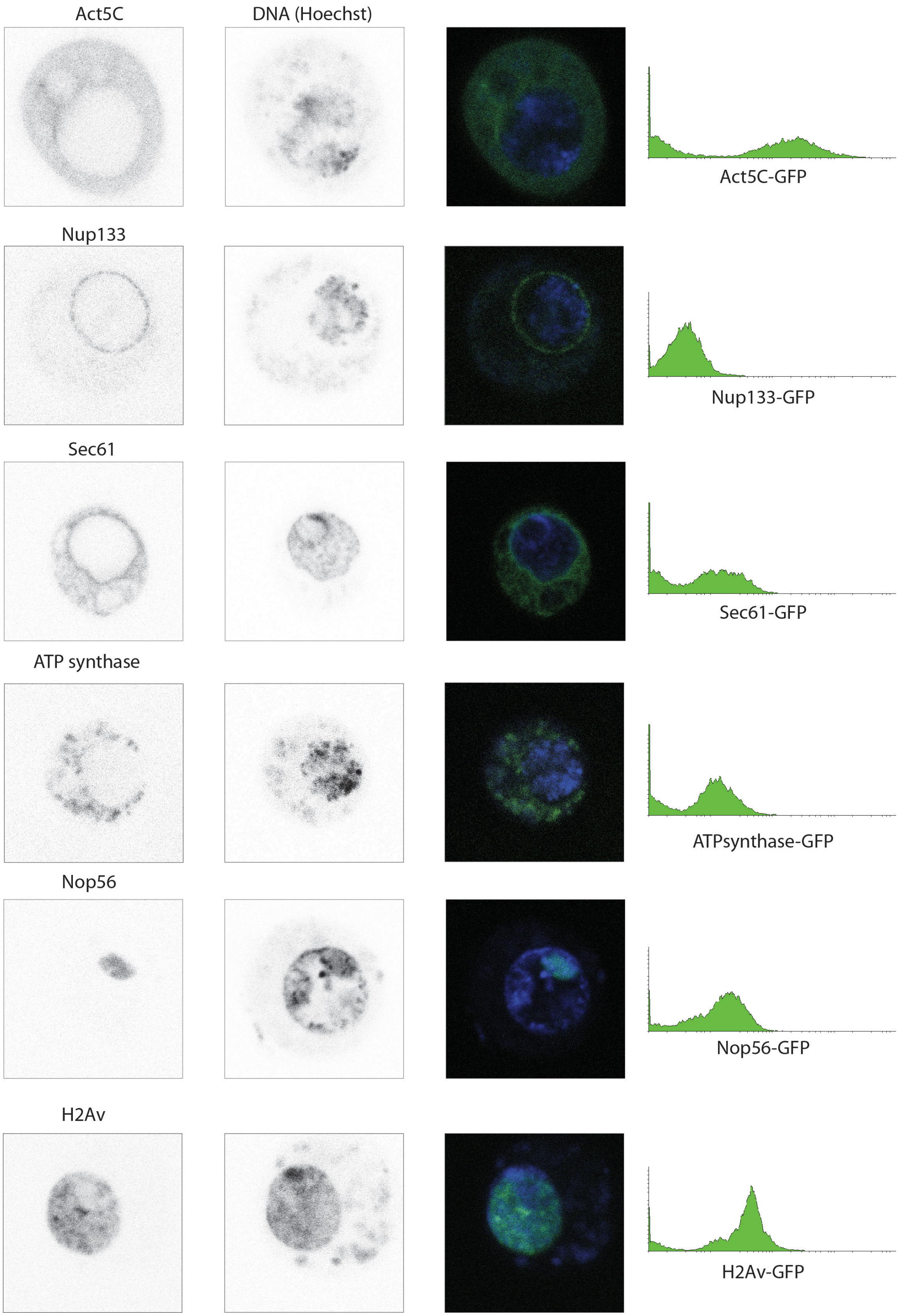

Figure S1:

Live cell confocal images of the GFP-tagged factors from Fig. 2 B in the manuscript; DNA was counter-stained with Hoechst 33342. A representative flow cytometry histogram is depicted on the right.
