# Supplementary material for: A Comprehensive Toolbox for Genome Editing in Cultured Drosophila melanogaster Cells
Source: G3 (Bethesda). 2016 Apr 13;6(6):1777–85. doi: 10.1534/g3.116.028241 (PMC4889673; doi:10.1534/g3.116.028241)
Supplement: Supplemental Material [file supp_g3.116.028241_FigureS2.pdf]

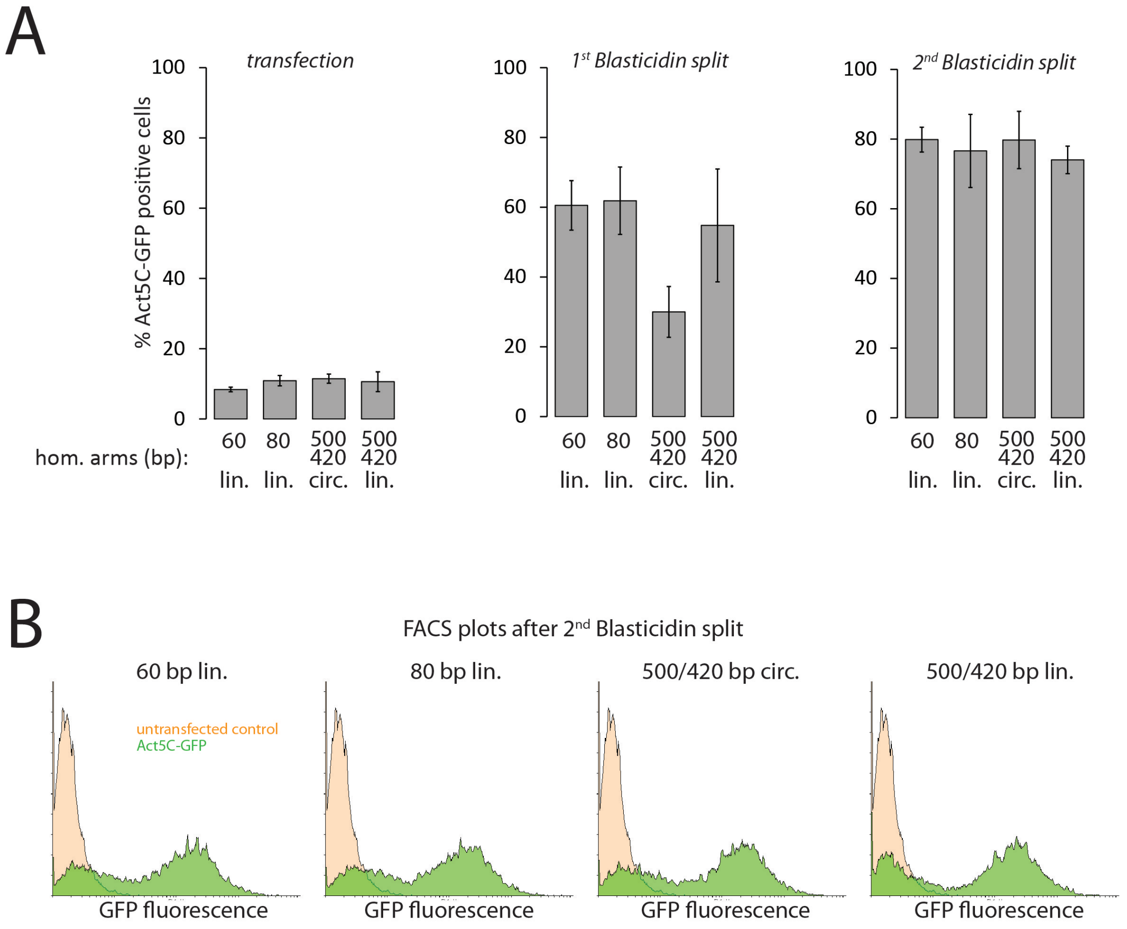

Figure S2: Comparison of long and short homology arms

A) Quantification of GFP-positive cells by flow cytometry before, during and after selection. The circular targeting vector is likely more stable in the cells after transfection and renders all transfected cells transiently Blasticidin resistant. This explains the lower frequency of Act5C-GFP positive cells after the first Blasticidin-split. Selection is nonetheless complete also for this sample after the second split.

B) Representative flow cytometry histograms comparing untransfected and selected cells from genome editing with the indicated homology arm length
