# Supplementary material for: A Comprehensive Toolbox for Genome Editing in Cultured Drosophila melanogaster Cells
Source: G3 (Bethesda). 2016 Apr 13;6(6):1777–85. doi: 10.1534/g3.116.028241 (PMC4889673; doi:10.1534/g3.116.028241)
Supplement: Supplemental Material [file supp_g3.116.028241_FigureS3.pdf]

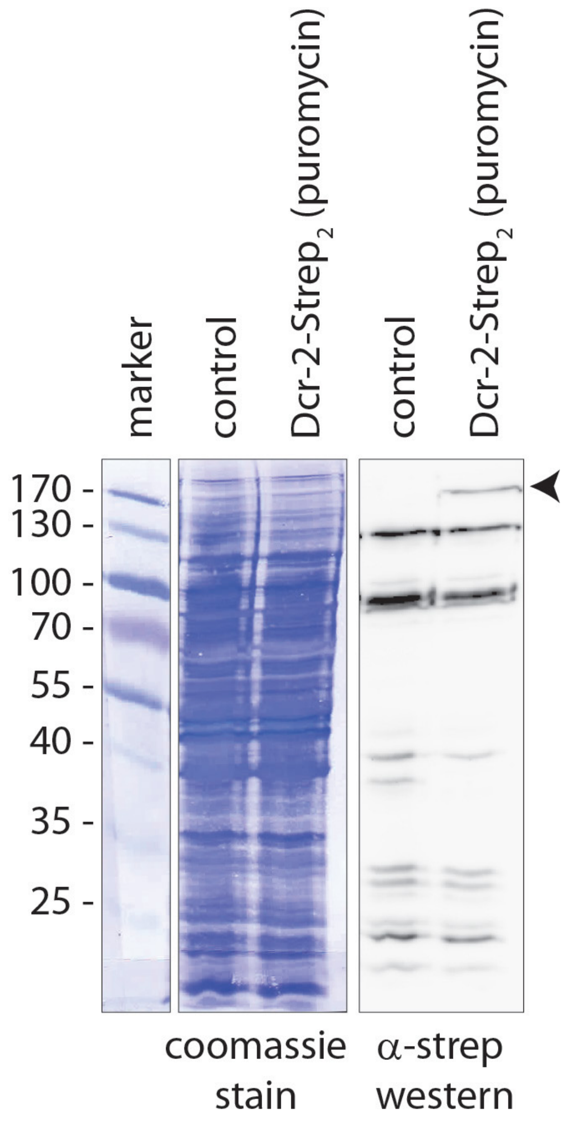

Figure S3: Genome editing with the puromycin resistance marker

As a proof-of-principle, we show the introduction of a C-terminal double-strep tag on the *dcr-2* gene; the tag is detected by western blot and the specific band is indicated with an arrowhead.
