# Supplementary material for: A Comprehensive Toolbox for Genome Editing in Cultured Drosophila melanogaster Cells
Source: G3 (Bethesda). 2016 Apr 13;6(6):1777–85. doi: 10.1534/g3.116.028241 (PMC4889673; doi:10.1534/g3.116.028241)
Supplement: Supplemental Material [file supp_g3.116.028241_FileS2.pdf]

Genomic Tagging in *Drosophila* cells

(N-terminal constructs)

Klaus Förstemann, Gene Center / LMU Munich
foerstemann@lmb.uni-muenchen.de

Version: Apr. 06, 2016

Contents:

Introduction 2
General considerations 8
A: Generation of Materials 9
A-1. Generation of a U6-sgRNA template for transfection by overlap-extension PCR 9
A-2. Generation of homologous recombination template for tagging by PCR 10
A-3. Generation of dsRNA to deplete *lig4* and *mus308* 11
B: Cell culture and transfection 13
B-1. Knock-down of *lig4* and *mus308* 13
B2. Transfection 14
B-3. Blasticidin or Puromycin selection of transfected cells 15
C: Molecular analysis 16
C-1. Molecular Analysis: Small-scale isolation of genomic DNA 16
C-2. Molecular Analysis: PCR to check for integration 17
C-3. Molecular Analysis: PCR to check for FLP-out of marker 19
C-4. Molecular Analysis: Western Blot 20
D: Cloning of cells expressing tagged proteins 21
References 22

Introduction

Repair of DNA double-strand breaks can occur via homologous recombination (normally with the replicated sister chromatid in mitotically dividing cells). This phenomenon can be exploited to manipulate the genome sequence experimentally with high precision. To this end, an artificial donor DNA molecule is provided. With a certain frequency, cells will use this homologous donor instead of the sister chromatid for repair. The specific introduction of a DNA double-strand break greatly stimulates the efficiency of HR-directed insertion of the desired DNA element. To this end, designer-nucleases (Zn-finger, TALEN) have been used with good success, but recently the CRISPR/cas systems have gained a lot of interest. The *cas9*-CRISPR system from *Streptococcus pyogenes* is particularly convenient since its sequence specificity can be programmed via an RNA subunit (derived from the CRISPR locus in *S. pyogenes,* see Fig. 1) [1-4]. This protocol describes a variation of our published approach for genome editing with PCR-based HR donor constructs [5].

The RNA subunit responsible for specificity can be expressed *in vivo* by creating a fusion gene between the U6 snRNA promoter (RNA polymerase III) and a DNA fragment encoding the CRISPR RNA. The construct can be designed at the computer and after oligonucleotide synthesis everything can be assembled in a single PCR via overlap extension (Figure 2).

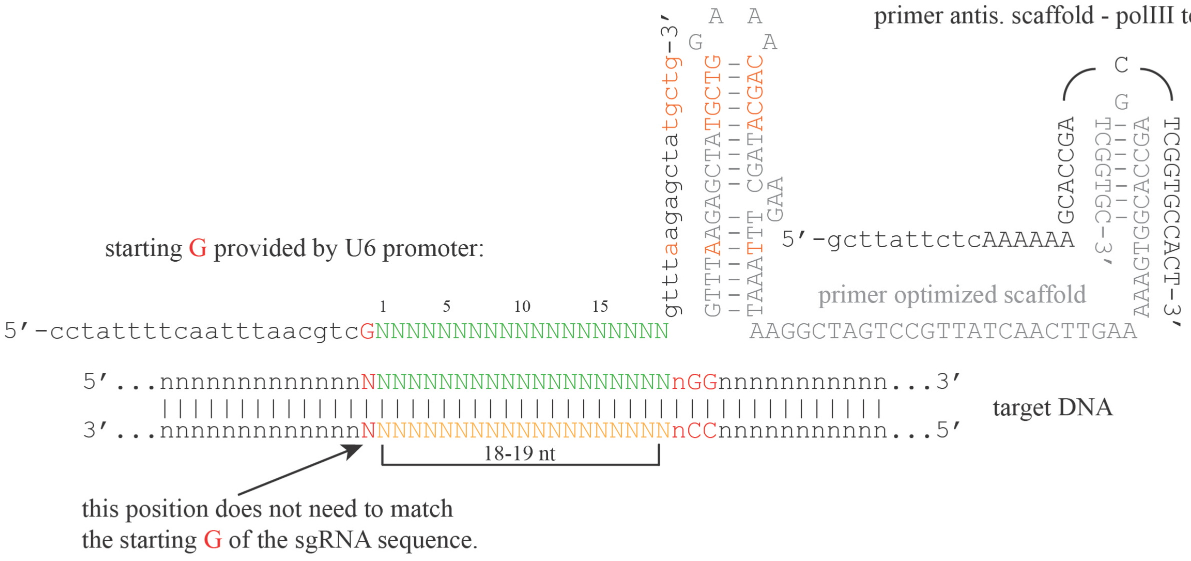

Figure 1:
Assembly of a DNA segment coding for the programming CRISPR RNA. The green sequence confers specificity for a target DNA locus. This sequence must be flanked by an NGG sequence (the protospacer associated motif = PAM) at the target locus in the genome; if that is the case then both strands of the target DNA will be cleaved by the *cas9* nuclease. 3′ of the programming "spacer" sequence, the scaffold RNA "repeat" is coded. Although the sequence depicted above is DNA, the resulting RNA secondary structure is indicated. 5′ of the programming sequence, a T7 RNA polymerase promotor sequence has been added (optional). This can be used for *in vitro* transcription or – as shown below – for overlap-extension PCR.

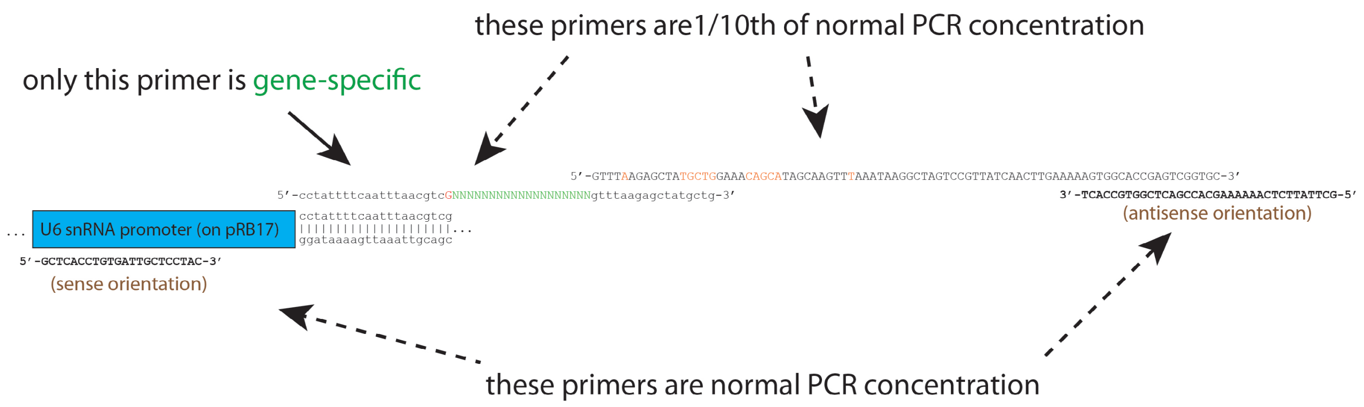

Figure 2:
Overlap extension PCR for U6-promoter CRISPR RNA fusions; the U6 snRNA promoter (~400 nt) has been cloned on plasmid pRB17 (with the sequence of the T7 RNA-polymerase promoter appended after the transcription start site, but we no longer use this feature). The sequence just upstream of the U6-promoter TSS is added to the oligonucleotide that provides the cleavage specificity sequence. (see detailed protocol). Assembly of the desired fusion gene occurs during PCR via the overlapping sequences. Only the outside primers are introduced at normal concentrations; the other oligonucleotides are more dilute, since their role is more the one of a PCR template rather than a primer.

To introduce a desired genomic modification at this locus, a template for homology-directed repair must be provided. This is referred to as the homologous recombination (HR) donor construct. To facilitate recovery of the desired events, a selection marker is introduced as well; it can later be removed by FLP-mediated site-specific recombination. The flanking homology regions needed in the HR donor construct can be introduced via cloning or simply added as extensions to PCR primers. For very short tags or point mutations, a synthetic single-stranded oligonucleotide can also serve as HR donor (not part of this protocol). Figure 3 illustrates the principles of each strategy.

Ideally, we choose a *cas9*/CRISPR target site that is disrupted upon integration of the tagging cassette. If a corresponding PAM sequence cannot be identified, the target site will also be present in one of the homology arms of the HR donor PCR product. In this case it is important to introduce a silent point mutation to prevent *cas9*/CRISPR mediated cleavage of the HR donor or the modified locus after integration.

In the case of an N-terminal tagging approach, an artificial promoter can be introduced to allow for expression of the targeted gene during selection (e.g. if full modification of an essential gene is desired). We chose the *mtnDE* promoter as this allows for inducible expression. In order to make this construct as small as possible (advantageous for PCR), the bidirectional activity of this promoter also drives expression of the Blasticidin-resistance. Thus, induction via $CuSO_4$ is mandatory during selection. We are also developing PCR template vectors that have constitutive expression of the resistance gene but inducible expression of the tagged gene (inquire if you are interested).

The tag is linked to the targeted protein via a short proline-alanin-serine stretch; this sequence is natively unstructured and thus flexible [6].

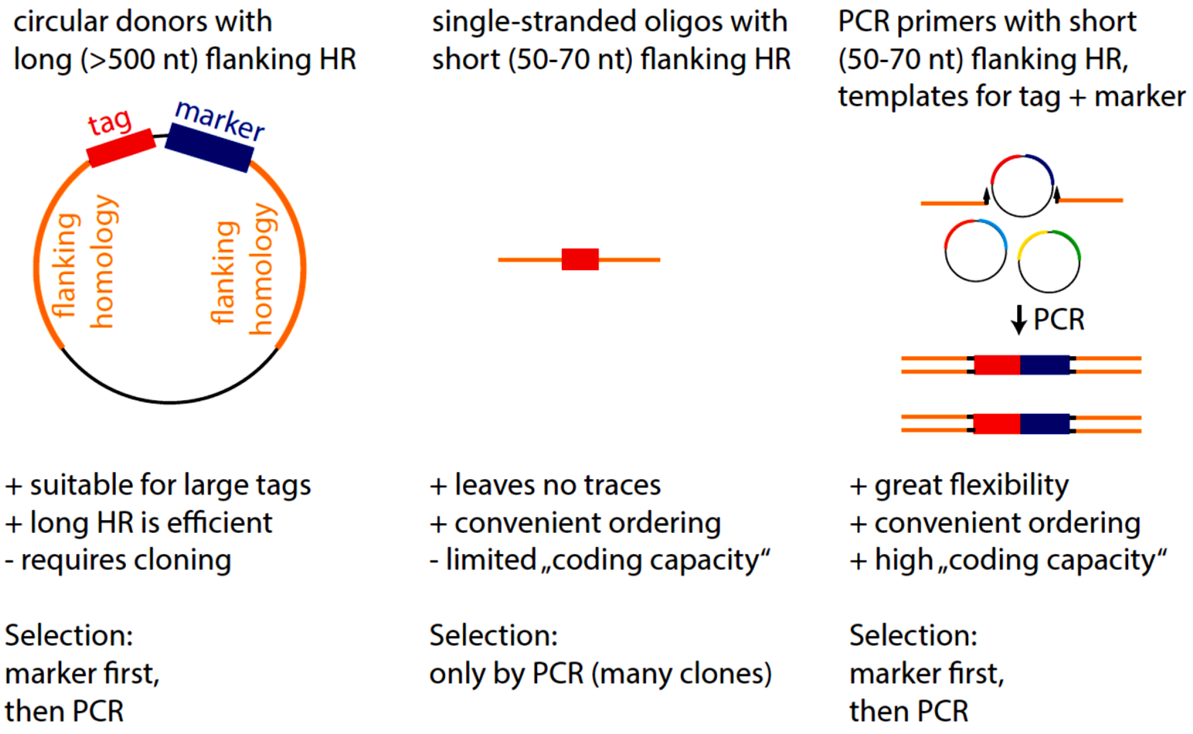

Figure 3:
Design principles of homologous recombination (HR) donor constructs. PCR selection refers to the test-PCR for integration of the construct at the desired genomic locus.

The synthesis of the HR donor constructs by PCR and design of the primers is described in figures 4, 5 and 6. This protocol is for N-terminal addition of a protein tag. Addition of a tag at the C-terminus is conceptually analogous but certain details change. To avoid confusions, we created a separate protocol for C-terminal tagging.

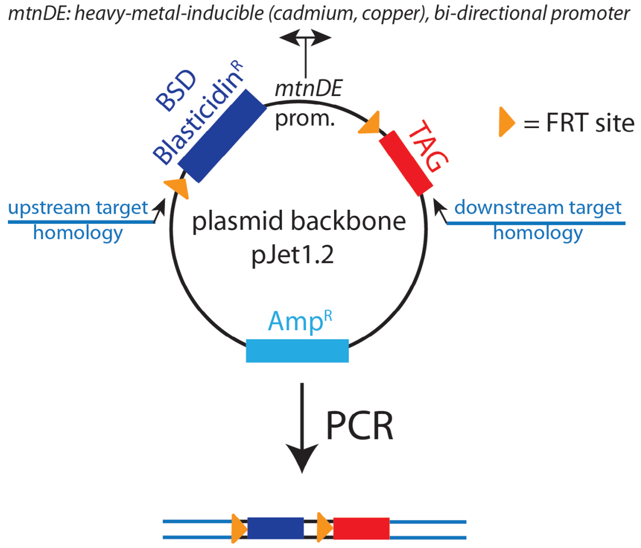

Figure 4:
Principle of HR donor generation for N-terminal tagging via PCR; note that until removal of the selection cassette via FLP-mediated recombination, the *mtnDE* promoter confers heterologous regulation on the targeted gene.

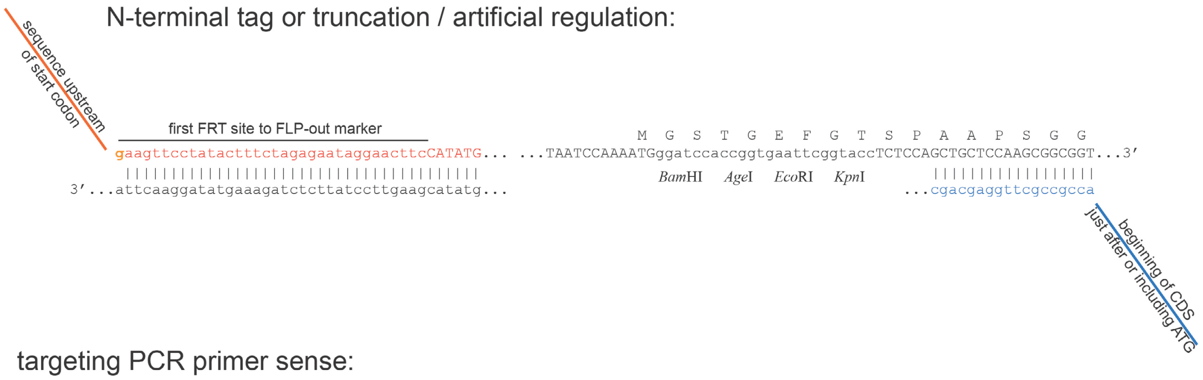

targeting PCR primer sense:

5'-nnnnnnnnnnnnnnnnnnnnnnnnnnnnnnnnnnnnnnnnnnnnnnnnnnnnnnnnnnnngaagttcctatactttctagagaataggaacttcCATATG-3'

sequence upstream of start codon, ***sense***
(we recommend using 60 nt here)

targeting PCR primer antisense:

5'-nnnnnnnnnnnnnnnnnnnnnnnnnnnnnnnnnnnnnnnnnnnnnnnnnnnnnnnnnnnnaccgccgcttggagcagc-3'

sequence downstream of start codon (ATG may also be included), ***antisense***
(we recommend using >=60 nt here)

Figure 5:

Details of primer sequences; the first FRT sequence deviates slightly from the desired sequence (likely a cloning artifact); this can easily be corrected via the sense primer used to generate the HR donor PCR product.

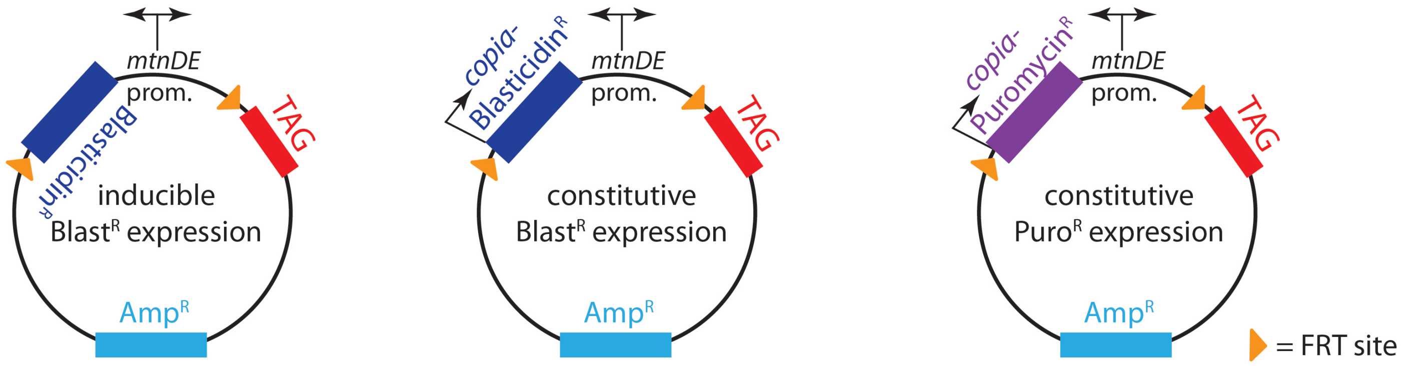

base vector:

```
                                                 M  G  S  T  G  E  F  G  T  S  P  A  A  P  S  G  G
...gaagttcctatactttctagagaatagg aacttcTAATCCAAAATGggatccaccggtgaattcggtaccTCTCCAGCTGCTCCAAGCGGCGGT...
         FRT site                                  BamHI  AgeI  EcoRI  KpnI
```

Flag$_3$ template:

```
                                                 M  G  S  T  G  E  F  D  Y  K  D  H  D  G  D  Y  K  D  H  D  I  D  Y  K  D  D  D  K  G  T  S  P  A  A  P  S  G  G
...gaagttcctatactttctagagaataggaacttcTAATCCAAAATGggatccaccggtgaattcGACTACAAGGACCACGACGGCGACTACAAGGACCACGACATCGACTACAAGGACGACGACAAGggtaccTCTCCAGCTGCTCCAAGCGGCGGT...
         FRT site                                  BamHI  AgeI  EcoRI             3xFLAG-tag                             KpnI
```

1x Strep template:

```
                                                 M  G  S  S  A  W  S  H  P  Q  F  E  K  T  G  E  F  G  T  S  P  A  A  P  S  G  G
...gaagttcctatactttctagagaataggaacttcTAATCCAAAATGggatccAGCGCTTGGAGCCACCCGCAGTTCGAAAAAaccggtgaattcggtaccTCTCCAGCTGCTCCAAGCGGCGGT...
         FRT site                                  BamHI        STREP-tag          AgeI  EcoRI  KpnI
```

Strep-His-Strep-TEV template:

```
                                                 M  G  S  S  A  W  S  H  P  Q  F  E  K  G  G  H  H  H  H  H  H  H  H  G  S  W  S  H  P  Q  F  E  K  T  G  E  F
...gaagttcctatactttctagagaataggaacttcTAATCCAAAATGggatccAGCGCTTGGAGCCACCCGCAGTTcgagaaaggtggaCATCACCACCATCACCATCATCACggatcgtggagccacccgcagttcgaaaaaaccggtgaattc
         FRT site                                  BamHI        STREP-tag             His8-tag                   STREP-tag          AgeI

                                    E  N  L  Y  F  Q ↓ G  E  F  G  T  S  P  A  A  P  S  G  G
          (continued from above)    GAAAACCTGTATTTTCAGGGCgaattcggtaccTCTCCAGCTGCTCCAAGCGGCGGT...
                                       TEV-cleavage site    KpnI
```

GFP template:

```
                                                 M  G  S [M  V                    Y  K] E  F  G  T  S  P  A  A  P  S  G  G
...gaagttcctatactttctagagaataggaacttcTAATCCAAAATGggatccATGGTG...     (717 nt eGFP)     ..TACAAGgaattcggtaccTCTCCAGCTGCTCCAAGCGGCGGT...
         FRT site                                  BamHI                                    EcoRI  KpnI
```

Tag	Blasticidin-resistance		Puromycin-resistance		inducible Blasticidin	
	name	*Addgene-#*	*name*	*Addgene-#*	*name*	*Addgene-#*
**base vector**	*pRB36*	72861	*pRB35*	72866	*pKF292*	72856
**3xFlag**	*pRB34*	72862	*pRB33*	72867	*pKF293*	72857
**Strep**	*pRB32*	72863	*pRB31*	72868	*pKF294*	72858
**Strep-His$_8$-Strep-TEV**	*pRB38*	72864	*pRB37*	72869	*pKF290*	72859
**eGFP**	*pRB40*	72865	*pRB39*	72870	*pRB30*	72860

Figure 6:
With a single set of target homology containing primers, a series of HR donor products can be generated by exchanging the template plasmid. The template vectors are available at Addgene.

Ideally, the HR donor construct integrates via homologous recombination at the desired locus. Alternatively, non-homologous end joining (NHEJ) can also lead to genomic integration of the donor construct; in this case the integration site is not directed but random. To reduce these unwanted NHEJ-events, transient depletion of the essential NHEJ-factor Lig4 via RNAi has proven effective (see Fig. 7). When this knock-down is combined with a knockdown for the microhomology-meiated end joining pathway enzyme DMA polymerase theta (*mus308*), a further increase in efficiency can be achieved.

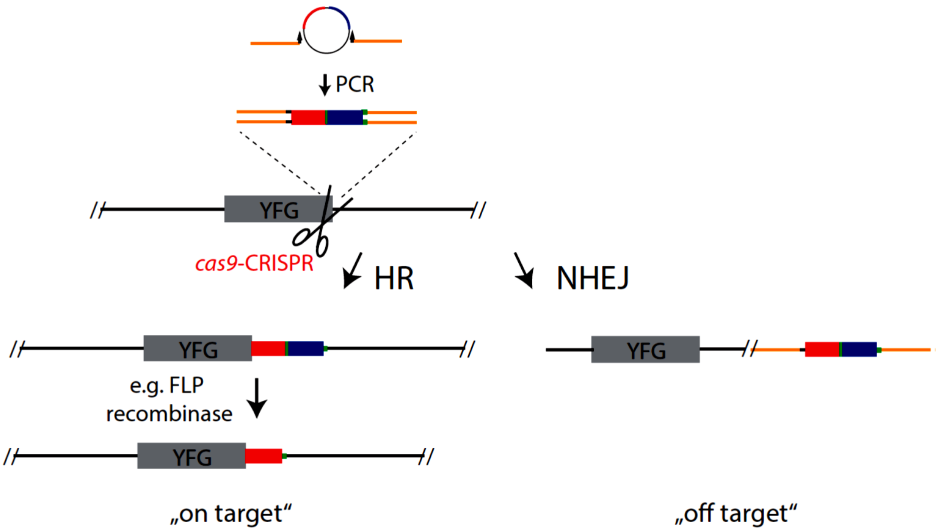

Figure 7:
Non-homologous end-joining competes with targeted integration; it can be reduced by RNAi or inhibition of *lig4* and *mus308* (the latter is essential for microhomology-mediated end joining MMEJ).

General considerations

1. PCR conditions

The PCR programs (cycling protocols) given in this protocol have been established in our lab on our thermal cyclers (Eppendorf Mastercycler Gradient, Sensoquest Labcycler Gradient). Depending on you model of cycler and PCR tubes, it is possible that you may have to adapt/optimize the conditions in order to get enough product.

2. Thermostable polymerase

None of the proofreading PCR polymerases that we tried so far (Pfu, Phusion, Q5) have yielded sufficient amounts of the desired product when used on their own. Standard Taq polymerase, however, is a reliable partner for the reactions needed. In our experience, a 1:1 mix of Taq and Pfu polymerase also works well. We hope that this mixture may display a somewhat reduced error rate during PCR, but we have not tested this specifically. As mentioned above, the Pfu polymerase does not give satisfactory results when used alone. Thus, its contribution to the final product is likely limited.

Expression plasmids are available at Addgene for Taq (pAKTaq, #25712) and Pfu (pET16B.Pfu, #12509). The enzymes are quite easy to prepare.

3. PCR buffers

Many commercially provided PCR buffers contain some detergent to stabilize the polymerase protein. We found that this has a negative effect on transfection efficiency, thus we recommend purification of the PCR product (e.g. via the Qiagen PCR purification kit) in this case. Alternatively, it is easy to make a detergent-free 10x PCR buffer (500 mM KCl, 100 mM Tris-HCl pH 8.3 at room temperature). In our experience, a PCR that was performed in this buffer can be directly transfected with good efficiencies. However, the yield in PCR may be slightly lower and you may have to "fiddle around" more in order to get the reaction to work in your lab.

4. Oligonucleotide synthesis

The longer the oligonucleotide, the more expensive it is - this logic is of particular importance for the HR donor PCR primers. We have thus tried to use shorter homology-containing regions and found that in principle it is possible to make the primers shorter, but it does reduce the efficiency of HR [5]. In other words, you will recover a lower proportion of drug-resistant cells that express the tag of interest. We buy our oligonucleotides at Eurofins/MWG (http://www.eurofinsgenomics.eu/), they offer the synthesis of primers with a maximal length of 120 nt. Other suppliers can probably provide equally suited reagents, we recommend running a 15% Urea/PAGE gel (standard mini-gel size as for Western blots is sufficient) to compare the quality of long primers between suppliers. It can be stained with Sybr Gold or ethidium bromide, loading 1 µl of a 1 µM solution is amply sufficient. We order the primers without any additional purification (i.e. no HPLC or PAGE purification, despite the website recommendation) and find that this works well for tagging while keeping the price in a very reasonable range. However, we have not made a quantitative comparison between standard and purified primers.

A: Generation of Materials

A-1. Generation of a U6-sgRNA template for transfection by overlap-extension PCR

Material:

- general PCR reagents
- sgRNA optimized scaffold [1, 7] primer serving as template during PCR (1 µM concentration):

`5′-GTTTAAGAGCTATGCTGGAAACAGCATAGCAAGTTTAAATAAGGCTAGTCCGTTATCAACTTGAAAAAGTGG CACCGAGTCGGTGC-3′`

- U6 promoter sense primer for PCR (10 µM concentration)

`5′-GCTCACCTGTGATTGCTCCTAC-3′`

- sgRNA antisense primer for PCR with RNA pol-III termination signal (10 µM concentration)

`5′- gcttattctcAAAAAAGCACCGACTCGGTGCCACT-3′`

- Sense primer for PCR containing the specific sequence for programming of the cas9 nuclease (1 µM concentration, specific sequence is underlined and in upper case letters – use 18 or 19 nt)

e.g. to target the *tub56D* gene:

`5′- cctattttcaatttaacgtcgCTAAAACAAATACAAAATGgtttaagagctatgctg-3′`

<u>PCR mix for sgRNA template:</u>

2	µl	1 uM oligo sgRNA scaffold
2	µl	1 uM primer CRISPR
2	µl	10 ng/µl plasmid pRB17 = U6-promoter fused to T7 promoter (Addgene #52527)
2	µl	10 uM primer U6-promoter sense
2	µl	10 uM primer sgRNA antisense
10	µl	10x PCR buffer without detergent (10x conc.: 500 mM KCl, 100 mM Tris-HCl pH 8.3)
16	µl	25 mM $MgSO_4$
2	µl	10 mM (each) dNTP
60	µl	H_2O
2	µl	Taq/Pfu polymerase mix (1:1)

<u>PCR program:</u>

1:	94°C	2min.
2:	94°C	30 sec.
3:	50°C	30 sec.
4:	72°C	30 sec.
5:	goto	step 2, 34 repetitions
6:	4°C	pause

The use of a PCR buffer without detergent makes it possible to directly transfect the PCR product without any purification. If you use a PCR buffer with detergent (may give higher yield), we recommend using a column-based PCR cleanup kit before transfection.

Quality control: Load 3 µl of the reaction on a 1.2% agarose gel. The band should run at ~600 nt.

A-2. Generation of homologous recombination template for tagging by PCR

Material:

- general PCR reagents
- template plasmids for tagging (100 pg/µl dilution)
- sense and antisense primers containing the desired homology regions (10 µM concentration); the length of the homology region can be varied according to the specification of your oligonucleotide synthesis provider. We recommend using at least 60 nt on the sense and the antisense primer.

e.g. sense for the *tub56D* gene gene (homology region in upper case letters):

```
5'- ACTGCTATAAGCGAAAGATAATTTTAAGTTAAAAGTTCAAAAGCCTAAAACAAATACAAAgaagttcctata
ctttctagagaataggaacttccatatg-3'
```

e.g. antisense for the *tub56D* gene (homology region in uppercase letters):

```
5'-ACTTACCTTGGCGCCGATTTGGTTGCCGCACTGACCAGCTTGGATGTGAACGATTTCCCTaccgccgcttgg
agcagctggaga-3'
```

PCR mix for HR-donor tagging PCR:

6	µl	100 pg/µl template plasmid
2	µl	10 uM primer sense
2	µl	10 uM primer antisense
10	µl	10x PCR buffer without detergent (10x conc.: 500 mM KCl, 100 mM Tris-HCl pH 8.3)
6	µl	25 mM $MgSO_4$
2	µl	10 mM (each) dNTP
70	µl	H_2O
2	µl	Taq/Pfu polymerase mix (1:1)

PCR program:

1:	94°C	2min.
2:	94°C	30 sec.
3:	50°C	30 sec.
4:	72°C	2 min.
5:	goto	step 2, 24 repetitions (=25 cycles in total)
6:	4°C	pause

The use of a PCR buffer without detergent (recipe below) makes it possible to directly transfect the PCR product without any purification. If you use a PCR buffer with detergent (may give higher yield), we recommend using a column-based PCR cleanup kit before transfection.

The reduced number of PCR cycles may lead to lower error rates; this potentially increases the recovery of correctly modified genomes, but we have not systematically tested this yet. However, this also reduces product yield. If more material is needed, you may either scale up the reaction volume or increase the cycle number to 30.

Hint: The U6-sgRNA fusion PCR (A-1) and the HR donor PCR (A-2) can also be performed in parallel in the same block. In this case, the HR donor cycling protocol should be used and 30 cycles are preferable.

Quality control: Load 3 µl of the purified product on a 0.8% agarose gel. The size of the amplicons should be between 1000 bp and 2000 bp, depending on template vector used.

A-3. Generation of dsRNA to deplete *lig4* and *mus308*

Material:

- general PCR reagents
- Primers with T7 extension (sense and antisense, 10 µM concentration) for *lig4*

sense: `5'-taatacgactcactatagggCCCAATGATCCAAAGTGTTTTTGCA-3'`

antisense: `5'-taatacgactcactatagGGAAGTAGGATGCCTTCGCGA-3'`

- Primers with T7 extension (sense and antisense, 10 µM concentration) for *mus308*

sense: `5'-taatacgactcactataggGCTGGGACTCCACCGGAAAG-3'`

antisense: `5'-taatacgactcactatagggTACCGTCGCCGTCCAGTAATG-3'`

- Drosophila cDNA as template for PCR
- 10x IVT buffer as described below
- 100 mM solution of each NTP — These can either be purchased ready-made or prepared from the powder, which is significantly cheaper. You need to adjust the pH to ~7 with NaOH (sticks are fine and help to avoid RNase contamination) after dissolving the salts!
- 1M DTT, RNase inhibitor, T7 RNA polymerase, RNase-free DNase-I

<u>PCR mix for dsRNA template (make separate reactions for *lig4* and *mus308*):</u>

1	µl	Drosophila cDNA
1	µl	10 uM primer sense
1	µl	10 uM primer antisense
5	µl	10x PCR buffer (we use Fermentas)
4	µl	25 mM $MgCl_2$
1	µl	10 mM (each) dNTP
36.5	µl	H_2O
0.5	µl	Taq polymerase

<u>PCR program:</u>

1:	94°C	2min.
2:	94°C	30 sec.
3:	50°C	30 sec.
4:	72°C	30 sec.
5:	goto	step 2, 34 repetitions (=35 cycles in total)
6:	4°C	pause

Use this product directly as template for *in vitro* transcription.

<u>10x T7 transcription buffer:</u>

500 mM	Tris-HCl pH7.9
25 mM	spermidine
260 mM	$MgCl_2$ (really 260 mM)
0.1%	Triton-X100

IVT recipe:

25 µl	PCR product from above
10 µl	10 x T7 transcription buffer
4 µl	ATP (100 mM)
4 µl	GTP (100 mM)
4 µl	UTP (100 mM)
4 µl	CTP (100 mM)
0.6 µl	1 M DTT
42.4 µl	H_2O (RNase-free)
4 µl	T7 RNA polymerase

IVT incubation

37°C	>= 3 hours, best is over night;	we use our incubator for bacterial plates for this as it will prevent condensation of water under the lid
add	1µl DNase-I	
37°C	30 min.	
95°C	10 min.	
65°C	20 min.	

Cool to RT, spin to remove the precipitate of Magnesium-pyrophosphate that may have formed. Make several aliquots of the supernatant and determine the concentration as described below.
This dsRNA can be added directly to the cell culture medium to induce RNAi by soaking, provided it is sterile (the 5 min. 95°C incubation described above is normally sufficient to sterilize the preparation).

Quality control: Load 1 µl and 1 µl of a 1:10 dilution on a 1% agarose gel. The band should run at ~600 nt, sometimes we see a certain extent of “laddering” (=multimer sized fragments). Ideally, the 1:10 dilution should still give a well visible band that can be used to estimate the concentration. Spectrophotometric quantification is impossible due to the presence of high amounts of free nucleotides.

B: Cell culture and transfection

B-1. Knock-down of *lig4* and *mus308*

Material:

- reagents prepared in section A of this protocol
- Fugene HD transfection reagent (e.g. Promega #E2311)
- Schneider's medium (we use Bio&Sell # BS 2.43G02J)
- Fetal bovine serum (FBS) and, if desired, Penicillin/Steptomycin as a cell culture additives; we prepare our medium with 10% FBS and 1x Pen/Strep.

Day 1: Split a dense culture of S2-cells to a density of *1x10^6 cells/ml* in Schneider's medium with 10% FBS; one well of a 24-well plate (= 500 µl of medium) is usually sufficient for about 10-15 editing transfections later. Add dsRNA for induction of *lig4* and *mus308* knock-down to a final concentration of 1 µg/ml.
Note: The combined knock-down of the two end-joining pathways is synthetically sick/lethal in S2-cells. If you observe that the cells are sick after the knock-down, reduce the dsRNA dose until the procedure is well tolerated.

Day 4: Count the cells and adjust them to a density of *1.5 x 10^6 cells/ml* in Schneider's medium with 10% FBS. Dispense 80 µl of this cell suspension per well of a 96-well plate. The cells are now ready for transfection. Alternatively, you can assemble the transfection mix in the plate first, then add the cell suspension to the mix (= reverse transfection).
Note: If you prefer a larger format for your transfections, scaling up the volumes according to the total culture volume is a good start. For example, in a 24-well dish you may use a total culture volume of 500 µl; you should thus plate 400 µl of cell suspension and use a total volume of 100 µl for the transfection mix.

For maximum convenience and efficiency, use cells with stable *cas9* expression such as our S2-cell clones 9-4 (neomycin-resistant) or 5-3 (hygromycin-resistant) with myc-tagged *cas9*.

B2. Transfection

Prepare the transfection mix (amount given per well) according to the type of experiment you are doing (transient or stable *cas9* expression). We find that the procedure is most efficient with stable cas9 expression; transient co-transfection of a *cas9* expression plasmid leads to a slightly lower proportion of tag-expressing cells after selection and growth recovery of the culture during selection is slower. The amounts of Fugene-HD and nucleic acids may need to be adjusted according to your cells and culture conditions.
We recommend the Act5C-GFP tagging approach for optimization (see C-terminal tagging protocol).

A) Transient expression of cas9
10 µl Schneider's medium without FBS (or the required amount to reach a final volume of 20 µl)
50 ng of U6-sgRNA fusion PCR (step A-1)
50 ng of pRB14 (or any other plasmid that permits expression of a *cas9* enzyme in *Drosophila* cells)
50 ng of HR template PCR product (step A-2)
Mix, then add 1 µl Fugene-HD directly from the glass vial

This approach will likely work in any cultured and transfectable *Drosophila* cell.

B) Stable expression of cas9
10 µl Schneider's medium without FBS (or the required amount to reach a final volume of 20 µl)
75 ng of U6-sgRNA fusion PCR (step A-1)
75 ng of HR template PCR product (step A-2)
Mix, then add 1 µl Fugene-HD directly from the glass vial

Controls: It is a good idea to carry along a positive control (e.g. GFP-*tub56D*) and a negative control for your targeting construct. One suggestion for a negative control is to leave out the sgRNA component. Another negative control should be to leave out the HR donor PCR; this is at the same time a positive control for the Blasticidin- or Puromycin-selection later on.

Let the transfection mix stand for 60 minutes, then add the entire volume (20 µl) to the cell culture well (or prepare the mix in the wells, then add the cells). For larger transfections, scaling up the amounts of the reagents according to total culture volume is a good start.

B-3. Blasticidin or Puromycin selection of transfected cells

Material:
- Schneider's medium with 10% FBS
- Blasticidin-S solution 10 mg/ml (e.g. Life Technologies A11139-03)
- Puromycin solution 10 mg/ml (e.g. Life Technologies A11138-03)

Day 2 post transfection: when using the inducible Blasticidin marker, add $CuSO_4$ to a final concentration of 100 µM; this concentration suffices to express sufficient Blasticidin-resistance for selection. Note that this will also lead to the expression of the tagged gene (advantageous in the case of essential genes, problematic if a dominant-negative truncation is attempted). Use constructs based on constitutive marker expression if induction of the tagged gene is not desired at this point. If you are tagging an essential gene, induction of the tagged locus is mandatory if you aim for modification of all genomic alleles.

Day 4 post transfection: Split the cells 1:5 into medium containing 10 µg/ml Blasticidin (=50 µl of 10mg/ml stock for 50 ml of medium) or 0.5 µg/ml Puromycin (= 2.5 µl of 10mg/ml stock for 50 ml of medium) and 100 µM $CuSO_4$; the cells will proliferate rather slowly in the beginning.

Day 11 post transfection: Split the cells again 1:5 into 10 µg/ml Blasticidin or 0.5 µg/ml Puromycin and 100 µM $CuSO_4$ containing medium. This time, growth in Blasticidin medium should resume almost normally. Sometimes the cells have not proliferated enough by day 11; in this case, wait a few more days until you perform the second split.

Note: In our hands, selection is complete after this split. Molecular analysis (PCR, western Blot) can already be performed after one round of selection. Since this requires some more material, you may find it convenient to directly split the cells up from 96-well to 24-well format plates on day 4 post transfection.

Marker removal:
If you wish to remove the selection marker from the cells' genomes, you can transfect them with a FLP-recombinase expression plasmid (e.g. pKF295, Addgene #71872). This will delete the *copia*-blasticidin or puromycin resistance cassette and leave a single FRT-sequence as the editing "scar" (in addition to the tag-sequence, of course). Since FLP-out and transfection efficiencies are not 100%, you need to perform clonal selection.

C: Molecular analysis

C-1. Molecular Analysis: Small-scale isolation of genomic DNA

Unfortunately, one cannot directly use resuspended cells as templates for PCR (= the equivalent of a colony-PCR in microbiology) because of inhibitory effects (either in the medium or from the cells). Thus, it is best to make a small-scale DNA isolation if you have only a limited number of samples. See below for a direct-PCR recommendation if higher throughput is required.

Material:
- Gel-extraction kit (e.g. Qiagen)
- cultured cells (~50-100 ul)

Procedure:
1. Resuspend cells in culture vessel and transfer 100 ul (50 ul can suffice if the amount is limiting) to an Eppendorf tube
2. Add 300 ul of Qiagen buffer QG to the cells in medium, vortex thoroughly
3. Apply the entire sample to a Qiagen spin column from the gel extraction kit and discard the flow-through
4. Wash the column with 700 ul of buffer PE and discard the wash
5. Centrifuge the empty column 1 min. at full speed to completely dry the matrix
6. Transfer column to fresh Eppendorf cup; apply 50 ul of buffer EB to the column, let stand for 1 min., then centrifuge for 1 min. at full speed to recover the eluate

This DNA preparation works well as template in PCR.
Use 2 µl of DNA per 25 µl reaction.

Although this procedure is reasonably fast and convenient, it is labor-intensive and costly if a large number of e.g. cell clones needs to be analyzed. In this case, the use of a self-made PCR buffer with high pH (~9.0-9.5) [8] can overcome the inhibitory effect of resuspended cells and medium, albeit at the cost of an overall reduction in PCR efficiency. We have made good experience using this approach for screening cultures, but you should limit it to short amplicons (e.g. use primer #452 from Fig. 8). The PCR conditions may need to be further optimized depending on the gene specific primer. Thorough genotyping of selected clones is best done with purified genomic DNA.

C-2. Molecular Analysis: PCR to check for integration

Material:
- purified DNA from selected S2-cells (step C-1)
- general PCR reagents
- primer us_common_sense (#451 in Fig. 8): 5'-aataggaacttcTAATCCAAAATGgga-3'
- gene-specific downstream antisense primer (recommended: 100-500 nt downstream of integration site, antisense orientation)

Procedure:
1. Assemble components of the PCR on ice except the Taq polymerase, use 2.5 µl template
2. Start thermocycler and set it to pause with the block at 94°C
3. Add Taq polymerase to PCR and close tube
4. Place tube from ice directly into the hot block of the thermal cycler, run PCR program
5. Analyze 10 µl of the PCR on a 1% agarose gel. See Fig. 8 for information on how to calculate the expected band size.

<u>PCR recipe:</u>

2	µl	isolated genomic DNA (or resuspended cells)
1	µl	10 uM primer us_common_sense
1	µl	10 uM primer gene specific antisense
2.5	µl	10x PCR buffer (standard pH with purified DNA template, high pH with resuspended cells)
3	µl	25 mM $MgCl_2$
0.5	µl	10 mM (each) dNTP
16	µl	H_2O
0.5	µl	Taq polymerase

<u>PCR program:</u>

1:	94°C	2min.
2:	94°C	20 sec.
3:	55°C	20 sec.
4:	72°C	90 sec.
5:	goto	step 2, 34 repetitions (=35 cycles in total)
6:	4°C	pause

Possible modifications:
- use a hot-start enzyme if you do not wish to preheat the thermocycler;

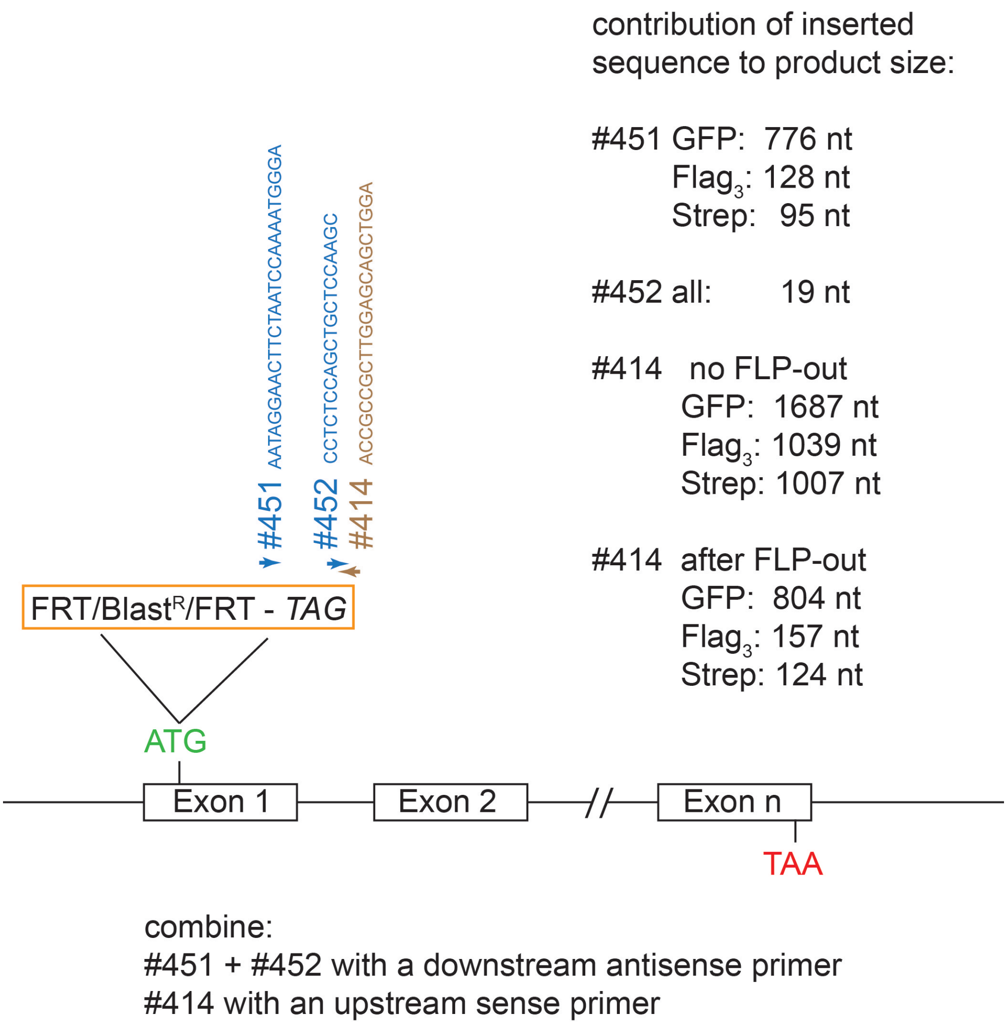

Figure 8:

This is a schematic drawing of the target locus and the integrated cassette. The oligonucleotide numbers refer to our lab's list; the sequences are given 5' to 3'. The size of the expected PCR product is calculated by adding the distance between the gene-specific primer and the integration site to the appropriate length as indicated in this figure. The primers will work for all selection cassettes; for PCR using primer #414 prior to marker FLP-out, the indicated sizes are for the inducible blasticidin resistance constructs; add ~250 nt for constitutively expressed blasticidin resistance and ~450 nt for constitutively expressed puromycin resistance.

C-3. Molecular Analysis: PCR to check for FLP-out of marker

This should be performed after transient transfection with pKF295 (Addgene #72871).

Material:
- purified DNA from selected S2-cells (step C-1)
- general PCR reagents
- primer ds_common as (# 414 in Fig. 8): 5′-ACCGCCGCTTGGAGCAGCTGGA-3′
- gene-specific upstream sense primer (recommended: 200-1000 nt upstream of integration site, sense orientation)

Procedure:
1. Assemble components of the PCR on ice except the Taq polymerase, use 2.5 µl template
2. Start thermocycler and set it to pause with the block at 94°C
3. Add Taq polymerase to PCR and close tube
4. Place tube from ice directly into the hot block of the thermal cycler, run PCR program
5. Analyze 10 µl of the PCR on a 1% agarose gel. See Fig. 8 for information on how to calculate the expected band size.

PCR recipe:

2	µl	isolated genomic DNA
1	µl	10 uM primer ds_common_as
1	µl	10 uM primer gene specific sense
2.5	µl	10x PCR buffer
2	µl	25 mM $MgCl_2$
0.5	µl	10 mM (each) dNTP
16	µl	H_2O
0.5	µl	Taq polymerase

PCR program:

1:	94°C	2min.
2:	94°C	20 sec.
3:	55°C	20 sec.
4:	72°C	90 sec.
5:	goto	step 2, 34 repetitions (=35 cycles in total)
6:	4°C	pause

Possible modifications:
- use a hot-start enzyme if you do not wish to preheat the thermocycler;

- the combination of gene-specific sense and antisense primers can also be used to check if all chromosomal alleles have been tagged. Since the unmodified locus will always produce a small PCR product, and is thus in a competitive advantage during PCR, any cell clone that produces only a product corresponding to the modified locus is very likely a clone with modification of all available alleles and thus offers heterologous expression control. This PCR only makes sense after clonal selection.

C-4. Molecular Analysis: Western Blot

Numerous Western blot protocols are available and whatever system is up-and-running in your lab will likely do the job. Therefore, this section is more a collection of items that you may find useful as additions or alternatives to established procedures. Note that the monoclonal Strep-Mab offered by IBA will only recognize the first instance of the Strep sequence in our Strep-His_8-Strep-TEV tag (Strep tag must be preceded by an alanine).

1) Sample preparations: For a quick check it is not necessary to make high-quality protein extracts; we harvest 50-100 µl of cells by centrifugation, aspirate the supernatant as much as possible to remove the serum-containing medium (consider using a suction device) and then resuspend & boil the cell pellet in 50 µl of 1x SDS sample buffer. Prior to loading, the samples are centrifuged for 5 minutes at top speed to pellet any non-solubilized material. This procedure generates quite reproducible and sufficiently concentrated extracts. Target proteins expressed at very low expression levels may nonetheless require some sort of specific extraction and concentration protocols prior to Western blot detection.

2) Transfer type: We routinely use a wet-transfer with Towbin buffer containing 10% Ethanol (instead of the 20% methanol) in a Bio-Rad chamber with electric tension set to 100 V. This gives good transfer results in 60 minutes, for proteins >100 kDa we increase transfer time to 75 or 90 minutes (this may require some optimization for each target protein).

3) Incubation with primary antibody: We dilute the primary antibodies (Flag: M2 / Sigma, GFP: B-2 / Santa Cruz, Strep: Strep-Mab HRP / IBA) 1:5000 in TBS with Tween-20 and 5% milk. For the anti-Flag M2, blocking/incubating/washing with 0.02% Tween gives excellent results, while for the GFP and Strep-Mab antibodies 0.1% Tween is preferable to avoid higher background staining. We find that incubation with the primary antibody overnight in a cold room (e.g. in a 50 ml conical tube on a roller) **significantly** increases signal strength compared with 120 minutes incubation at room temperature. The anti-Flag M2 monoclonal antibody also weakly recognizes one ~130 kDa and ~70 kDa endogenous *Drosophila* protein. Therefore, a control lane with untreated S2-cells is essential (and for the other tags a good idea, too).

4) Just a reminder if you are using the cell lines with stable cas9 expression: This construct has a myc-tag, therefore we do not recommend the use of myc-tag cassettes for other proteins in conjunction with our cell lines or vector for cas9-expression.

D: Cloning of cells expressing tagged proteins

Cell cloning can be done either with the Blasticidin-resistant population (only add copper to the medium if expression of the tagged gene is required for viability) or after the marker cassette has been FLP'ed out upon transient transfection of the pKF295 vector (Addgene # 72871).

1. Add 90 µl of selection media per well to a 96 well plate, leaving the first three colmuns empty. Including 20% of conditioned medium (= "old", sterile-filtered cell culture medium) usually improves the yield.
2. Count the cells that you are cloning and adjust the density to 8000 (=8x10^3) cells / ml. Dispense 120 µl of this into each well of the first three columns of the 96 well plate. This corresponds to plating ~1000 cells per well.
3. Using an eight channel multipipetter, transfer 30 µl from column 1 to column 4, from 2 to 5 and from 3 to 6. Then repeat this scheme for the rest of the plate. In the end, you have made three successive steps of 4-fold dilutions, with three columns (24 wells) per dilution step.
4. Wrap a strip of parafilm around the plate and incubate at 25°C for at least two weeks.

Note: Continue culturing the pool of selected cells in parallel in case no colonies develop.

5. Check wells to see if colonies are developing. You may even have to wait up to three weeks to see colonies by eye. If none have developed after three weeks, repeat selection with the pool of selected cells, starting with 5000 cells per well (i.e. ~ 4x10^4 cells/ml).
6. Note the position of isolated cell clumps with a waterproof pen on the bottom of the dish. This helps to find it again under the cell culture hood and to remove each colony in a maximum of 10 µl of medium with a pipette. First, transfer it to a fresh well of a 96-well plate containing 100 µl of medium.
7. When picked clones in the 96 well plate are getting dense, dilute them into 24 well plates. When these wells reach high density, split cells again and incubate the new plate as before. The old plate can now be used for molecular analysis as described in section C.
8. Amplify the clones of interest further in 6-well plates and freeze aliquots. To be really sure that the population is clonal (in cases where this is essential), we suggest performing two rounds of dilution & selection.

References

1. Mali, P., et al., *RNA-guided human genome engineering via Cas9.* Science, 2013. **339**(6121): p. 823-6.
2. Cong, L., et al., *Multiplex genome engineering using CRISPR/Cas systems.* Science, 2013. **339**(6121): p. 819-23.
3. Jinek, M., et al., *RNA-programmed genome editing in human cells.* eLife, 2013. **2**: p. e00471.
4. Jinek, M., et al., *A programmable dual-RNA-guided DNA endonuclease in adaptive bacterial immunity.* Science, 2012. **337**(6096): p. 816-21.
5. Bottcher, R., et al., *Efficient chromosomal gene modification with CRISPR/cas9 and PCR-based homologous recombination donors in cultured Drosophila cells.* Nucleic acids research, 2014. **42**(11): p. e89.
6. Schlapschy, M., et al., *PASylation: a biological alternative to PEGylation for extending the plasma half-life of pharmaceutically active proteins.* Protein engineering, design & selection : PEDS, 2013. **26**(8): p. 489-501.
7. Chen, B., et al., *Dynamic imaging of genomic loci in living human cells by an optimized CRISPR/Cas system.* Cell, 2013. **155**(7): p. 1479-91.
8. Bu, Y., H. Huang, and G. Zhou, *Direct polymerase chain reaction (PCR) from human whole blood and filter-paper-dried blood by using a PCR buffer with a higher pH.* Analytical biochemistry, 2008. **375**(2): p. 370-2.
